# Refractory density model of cortical direction selectivity: Lagged-nonlagged, transient-sustained, and On-Off thalamic neuron-based mechanisms and intracortical amplification

**DOI:** 10.1371/journal.pcbi.1008333

**Published:** 2020-10-14

**Authors:** Anton Chizhov, Natalia Merkulyeva

**Affiliations:** 1 Ioffe Institute, St.-Petersburg, Russia; 2 Sechenov Institute of Evolutionary Physiology and Biochemistry of RAS, St.-Petersburg, Russia; 3 Pavlov Institute of Physiology, St.-Petersburg, Russia; University of Pittsburgh, UNITED STATES

## Abstract

A biophysically detailed description of the mechanisms of the primary vision is still being developed. We have incorporated a simplified, filter-based description of retino-thalamic visual signal processing into the detailed, conductance-based refractory density description of the neuronal population activity of the primary visual cortex. We compared four mechanisms of the direction selectivity (DS), three of them being based on asymmetrical projections of different types of thalamic neurons to the cortex, distinguishing between (i) lagged and nonlagged, (ii) transient and sustained, and (iii) On and Off neurons. The fourth mechanism implies a lack of subcortical bias and is an epiphenomenon of intracortical interactions between orientation columns. The simulations of the cortical response to moving gratings have verified that first three mechanisms provide DS to an extent compared with experimental data and that the biophysical model realistically reproduces characteristics of the visual cortex activity, such as membrane potential, firing rate, and synaptic conductances. The proposed model reveals the difference between the mechanisms of both the intact and the silenced cortex, favoring the second mechanism. In the fourth case, DS is weaker but significant; it completely vanishes in the silenced cortex.DS in the On-Off mechanism derives from the nonlinear interactions within the orientation map. Results of simulations can help to identify a prevailing mechanism of DS in V1. This is a step towards a comprehensive biophysical modeling of the primary visual system in the frameworks of the population rate coding concept.

## Author summary

A major mechanism that underlies tuning of cortical neurons to the direction of a moving stimulus is still debated. Considering the visual cortex structured with orientation-selective columns, we have realized and compared in our biophysically detailed mathematical model four hypothetical mechanisms of the direction selectivity (DS) known from experiments. The present model accomplishes our previous model that was tuned to experimental data on excitability in slices and reproduces orientation tuning effects in vivo. In simulations, we have found that the convergence of inputs from so-called transient and sustained (or lagged and nonlagged) thalamic neurons in the cortex provides an initial bias for DS, whereas cortical interactions amplify the tuning. In the absence of any bias, DS emerges as an epiphenomenon of the orientation map. In the case of a biased convergence of On- and Off- thalamic inputs, DS emerges with the help of the intracortical interactions on the orientation map, also. Thus, we have proposed a comprehensive description of the primary vision and revealed characteristic features of different mechanisms of DS in the visual cortex with columnar structure.

## Introduction

Mathematical models of primary vision, aimed to be comprehensively comparable with experimental data are still under development. In the present paper we implement mechanisms of direction selectivity (DS) of primary visual cortex neurons into a model that has already shown its ability to reflect experimental data on cortical tissue excitability and orientation selectivity.

Visual information processing begins in the retina and subsequently passes through the visual thalamic structures (primarily the lateral geniculate nucleus, or LGN) to the primary visual cortex [1,2,3]. The neurons of the primary visual cortex are selective to various characteristics of a stimulus, such as orientation, the direction of motion, color, etc. [4,5,6]. Neurons preferring a particular orientation (orientational neurons) or direction (directional neurons) of a stimulus are unevenly distributed within the primary visual cortex and are grouped into the so-called functional columns or modules [7,8,9]. The columnar structure constitutes so-called hypercolumns, each including a full set of the orientational or directional columns. The functional maps with orientation hypercolumns have been found in tree shrews, ferrets, cats, and primates. Our modeling is focused to the visual cortex with the orientation hypercolumns. However, we also consider DS mechanisms revealed in rodents, for which experiments with intracellular recordings and optogenetic stimulation have been more developed.

Some important questions regarding the mechanisms underlying DS remain unanswered. One of the questions is whether DS is inherited from the directionally selective subcortical neurons (mainly geniculate cells) or is reconstructed in the cortex de novo. DS of geniculate cells is well-documented for lagomorphs and rodents [10], but data on carnivores and primates point to some directional bias in geniculate cells rather than a prominent DS [11,12]. Recently, Lien and Scanziani [13] used an optogenetic approach to show that even in mice, the cortical DS emerges de novo at the convergence of thalamic synapses on the same cortical neuron, whereas at the geniculate level, DS is negligible.

Most DS models include a time delay between the responses of the spatially shifted neighboring geniculate neurons terminating at the same cortical cell [14], similar to the mechanisms revealed in the retina [15]. These geniculate neurons reveal either an increase or a decrease in their activity in response to the light stimulus (so-called On and Off cells) or differences in the temporal characteristics of their responses. In the second case, LGN neurons are categorized as (i) transient and sustained (T-S), which generate fast- and slow-decaying responses, respectively [13,16,17], or (ii) lagged and nonlagged (L-N), depending on the latency to discharge after spot onset [18,19,20]. Such neurons exist in cats [17,19], monkeys [21,22], and mice [10] and have been proposed to provide the spatiotemporal offset for cortical DS [19,23].

The physiological mechanism of the delay formation was studied in several experimental works [24,25], and it has been demonstrated that the delay between responses to the center and surrounding area of a receptive field is determined by GABA-A (gamma-aminobutyric acid) receptors within the LGN. A complex model of DS has also been proposed based on specific convergent projections of the signals from LGN cells with and without a delay and intracortical interactions [26]. In this model, the temporal difference between the lagged and nonlagged cell responses and the structure of LGN inputs provide DS in V1. An alternative mechanism of DS based on T-S thalamic neurons converging on the same cortical neuron has been recently proposed by Lien and Scanziani [13]. The authors suggested that the T-S neurons generate a fast- and a slow-decaying excitatory postsynaptic current (EPSC), respectively, which combine into a compound EPSC. Thalamic neurons prefer distinct spatial phases, so the decay of the compound EPSC changes with the phase. Time-staggered summation results in large or small F1 modulations of the compound EPSC depending on the direction of the simulated motion. Thus, the DS of the cortical neuron depends on the phase shift between the T-S thalamic neurons.

Alternatively, recently reported experimental data obtained through the use of optogenetics [27] and multielectrode electrophysiological recordings [28] suggest that DS in V1 is determined by a displacement of On and Off subzones of the receptive fields of V1 neurons. However, a mechanism that is based on the On-Off subzone displacement and provides DS for symmetric white-and-black stimuli like gratings is unclear. Summarizing, some alternative mechanisms of DS have been discussed in the literature; however, the dominant mechanism is still unknown.

Besides of feedforward mechanisms, DS is highly dependent on the intracortical interactions, which follows even from the fact of weak correlation between the preferred directions evaluated from the thalamic input and the neuronal spike response [13]. The intracortical interactions provide some important effects of the primary cortex functioning, such as the prolonged responses to brief stimuli [29] or apparent motion [30]. As shown in experiments and models, the balanced intra-layer cortical interactions between inhibitory and excitatory populations play a major role in shaping the dynamic stimulus representations in the early visual cortex [31,32]. The question of interplay between the feedforward DS mechanisms and the intracortical interactions is an open question brought up in the present paper.

In our previous works, we extended our biophysically detailed, conductance-based population model of cortical orientation tuning [33,34]. This approach is alternative to comprehensive network approaches that simulate single neurons explicitly [35,36]. A single population is defined here as a large number of similar uncoupled neurons receiving similar inputs. In these notations, the cortex is considered a layered continuum of coupled populations. Due to the combination of (i) the population level description, which is optimal in the framework of the population coding of information, (ii) a quite accurate population approach, namely, a conductance-based refractory density (CBRD) method [37,38], and (iii) a hypercolumnar structure description of V1, the model was able to reproduce a vast series of experimental observations obtained in V1 in vitro and in vivo. In the present paper, we discuss the reproduction of the abovementioned three DS mechanisms using this model by relying on (i) lagged and nonlagged, (ii) transient and sustained, or (iii) On- and Off-neurons. Moreover, we explain whether DS may occur as an epiphenomenon due to the orientational hypercolumns in the absence of any thalamo-cortical bias. Using these four models, a potential role of intracortical interactions in DS has been identified by mimicking the silencing of the cortex.

## Results

**Fig 1:**
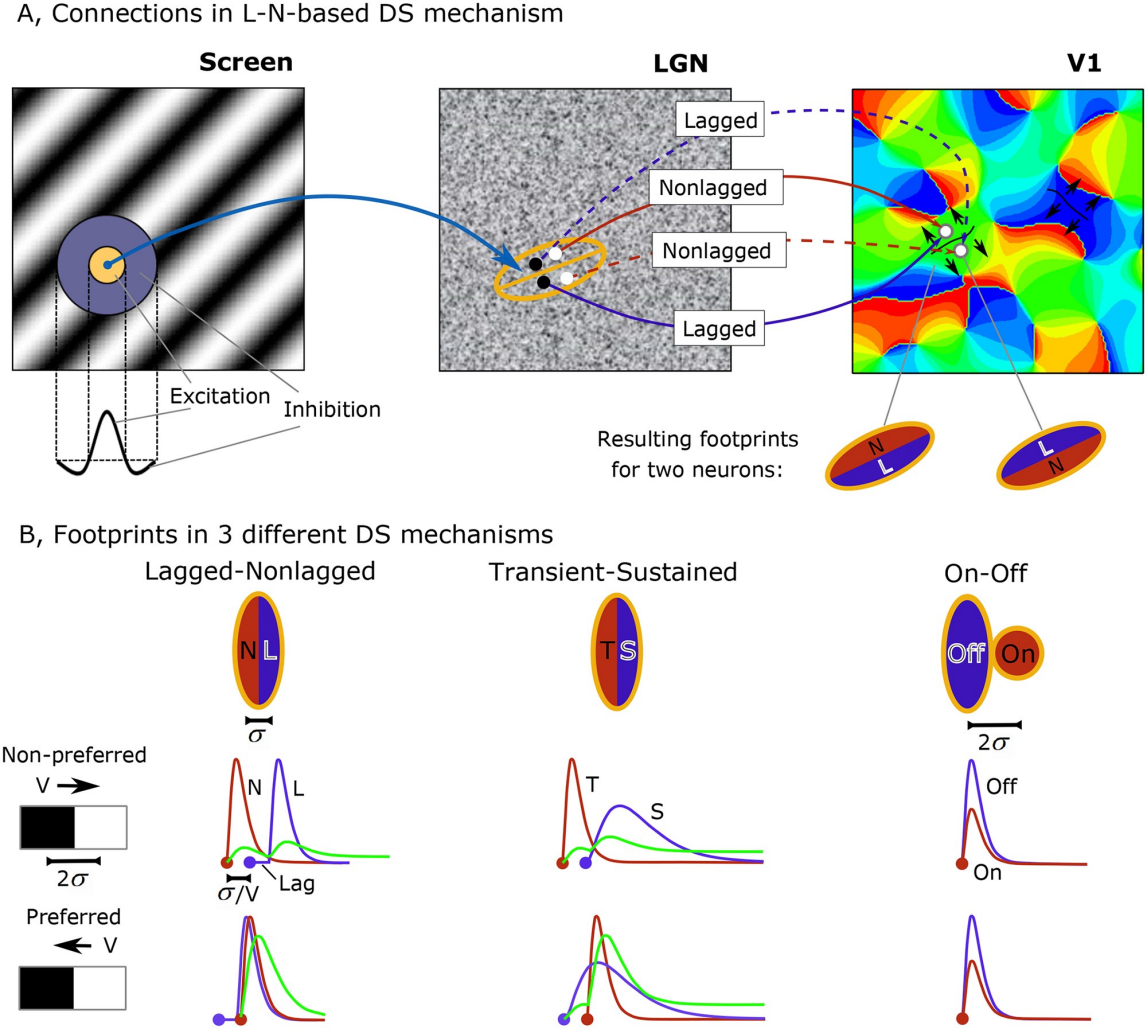
Schematic representation of neuronal connections in the mechanisms of directional selectivity (DS). A. An example of connections in the DS mechanism based on the lagged (L) and nonlagged (N) neurons. A V1 neuron that prefer upward direction (upper white dot on the right panel) receives thalamic input from the nonlagged cells (upper white dot on the middle panel and red arrow between the middle and right panels) located in the upper part of the footprint (red oval) and from the lagged cells (lower black dot on the middle panel and blue arrow between the middle and right panels) located in the upper part of the footprint. It is vice versa for the downward direction preferring neuron (lower white dot on the right panel). The orientation map was generated using the algorithm from [39] for the purpose of illustration. Speckled texture in LGN plot illustrates homogeneous distribution of lagged and nonlagged cells. B. Footprints in the three DS mechanisms: lagged-non-lagged, transient-sustained (T-S), and On-Off. Dots denote the time moments when the stimuli begin to excite the thalamic cells. (The initial stimulus is a gray screen). Sum of the inputs from the thalamic cells (red and blue curves) and a sigmoid-like nonlinearity of cortical neuron excitation determine the cortical cell activity (green), which depends on the direction of the stimulus. In the On-Off mechanism, the sum is identical, hence DS may derive only from nonlinear interactions within the orientation map. *σ* is the spatial scale in LGN, *V* is the stimulus speed, *Lag* is the characteristic delay of the L-neurons relative to N-neurons.

We present simulations performed with the model (see Methods), which combines the detailed, conductance-based description of neuronal population activity in area V1 with the simple, filter-based description of the retino-thalamic processing of visual signals. The generalized model retains the advantages of the former model [34], relating simulations to different experimental observations obtained in slices and *in vivo* in the visual cortex, and allowing the consideration of experiments with moving stimuli. These stimuli, such as moving gratings (Fig 1A, **left**) are used to calculate the fields of activity of the LGN neurons having center-surround receptive fields (RFs) (Fig 1A, **middle**), and the activity of the V1 area having neurons with more sophisticated RFs (Fig 1A, **right**). The elements are specific to each of DS mechanism.

According to the model, cortical neurons receive inputs from a particular “footprint” in the LGN; this footprint is a domain in the LGN that sends axons to a given V1 neuron [40]. This V1 neuron receives thalamic input only from that footprint domain of the LGN. The orientation and direction selectivity of cortical neurons are determined based on the properties of the LGN neurons and the structure of the footprint. The elongation of the footprint determines the cortical neurons’ orientation preference. DS depends on the asymmetry of the V1 neuron projections received from lagged (L) versus nonlagged (N), transient (T) versus sustained (S), or On- versus Off-neurons, for the L-N, T-S, or On-Off mechanisms of DS, respectively. Data on the asymmetry of those connections is lacking in the experimental literature, so for simplicity our simulation assumes that convergent L and N (or T and S) inputs to a cortical cell are spatially segregated, similar to the assumption about adjacent elongated T- and S-cell subfields made in the alternative modeling work [36]. In our simulations, the footprint of a V1 neuron splits into two halves along the axis of elongation, each sending signals from either lagged or nonlagged thalamic cells (Fig 1A, **right**). A similar footprint is taken for the T-S mechanism (Fig 1B, **middle**). The elongation of the footprints provides the orientation selectivity while their width determines the optimal wavelength of the stimulating gratings. For the On-Off mechanism, the footprint is shaped like the shifted, oval On- and round Off-subdomains (Fig 1B, **right**). The elongation of the On-subdomain provides an orientation selectivity even for the stimuli that evoke only the On-pathway. The magnitude of the shift effects the optimal wavelength of the stimulating gratings. This footprint structure is a prototype of that of typical RFs [28,41].

The sum of the inputs from the thalamic cells depends on the direction of the stimulus in the L-N and T-S mechanisms (Fig 1B). Together with a threshold-linear-like or sigmoid-like nonlinearity, this sum determines the cortical cell activity, which is significantly different for the preferred and non-preferred directions.

In the On-Off mechanism, the sum is identical for both directions, if the stimulus is as shown in Fig 1B (i.e. the displacement of the white and black subzones in corresponance to the displacement of the On- and Off-subfields of the footprint). This is also the case for the moving gratings of any wavelength. For gratings moving with a unity velocity, this issue can be clarified as follows. We can approximate the thalamic input through the On-subfield of the footprint as |sin(*t*)| and the Off-input as |sin(*t* + *π*)|, and we can introduce a ratio of Off-to-On contributions *k* and a subfield displacement Δ*φ*. The total input when the stimulus is moving in one direction is |sin(*t*)| + *k* |sin(*t* + *π* + Δ*φ*)|, and for the opposite direction, it is |sin(−*t*)| + *k* |sin(−*t* + *π* + Δ*φ*)|. The inputs have equal amplitudes for any *k* and Δ*φ*, which do not provide any preference for any direction. Thus, in the considered cases, DS cannot stem from just the subcortical projections, but it may derive only from nonlinear interactions within the orientation map.

### Tuning of the intracortical connections

**Fig 2:**
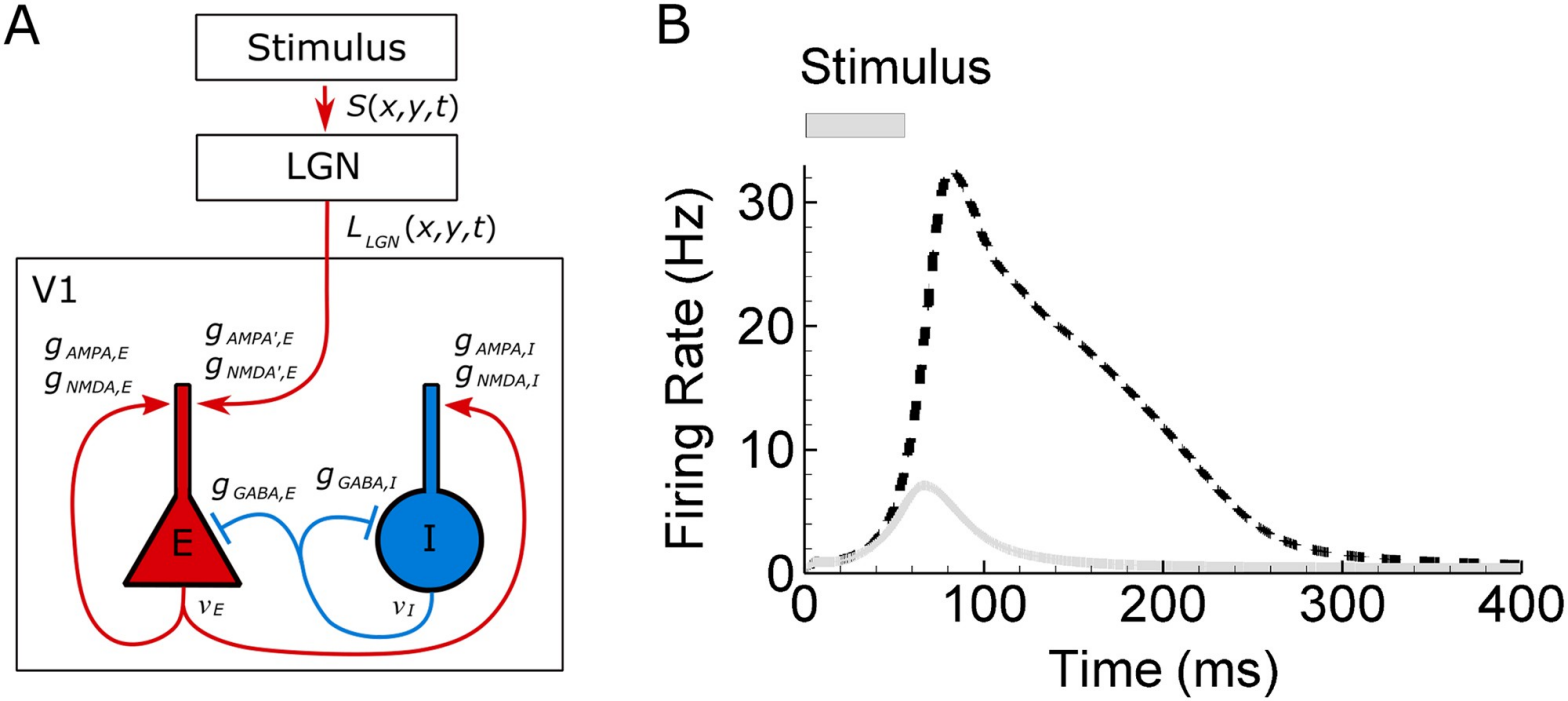
Schematic of connections (A) and long-lasting response to a short stimulus (B). Response to a round spot of light of the radius 0.5° and the duration 50ms. The response obtained with the default settings (dashed line) persists much longer than with the excitatory intracortical conductances reduced by 25% (solid gray).

Nonlinear interactions within the orientation map depend on the tuning of intracortical connections (Fig 2A). We believe that an important characteristic of recurrent connection tuning is the network ability to maintain its activity in response to a short stimulus. In experiments with monkeys [29], a short (50ms) spot of light evokes an activity that lasts more than 200 ms. A similar effect is observed in rodents and is shown to be provided by recurrent cortical excitatory circuits [42]. Our simulation of the response to a round spot produces results very similar to those of the experiment (Fig 2B). This effect is nontrivial because all the processes that provide positive feedback in the dynamical system under consideration are much slower than the response. The response is most sensitive to the recurrent excitatory connection strengths, ${\overset{-}{g}}_{AMPA,E}$ and ${\overset{-}{g}}_{NMDA,E}$, which reveals the role of intracortical recurrent excitation in maintaining the cortical activity. With all excitatory intracortical conductances reduced by 25% the response duration dramatically decreases. This effect will be a subject of future analysis as it is out of the scope of the present paper. However, here we explore the model with sharply tuned intracortical connections to study the effects of intracortical interactions on DS pre-determined by different thalamo-cortical footprints.

### Three mechanisms provide DS

**Fig 3:**
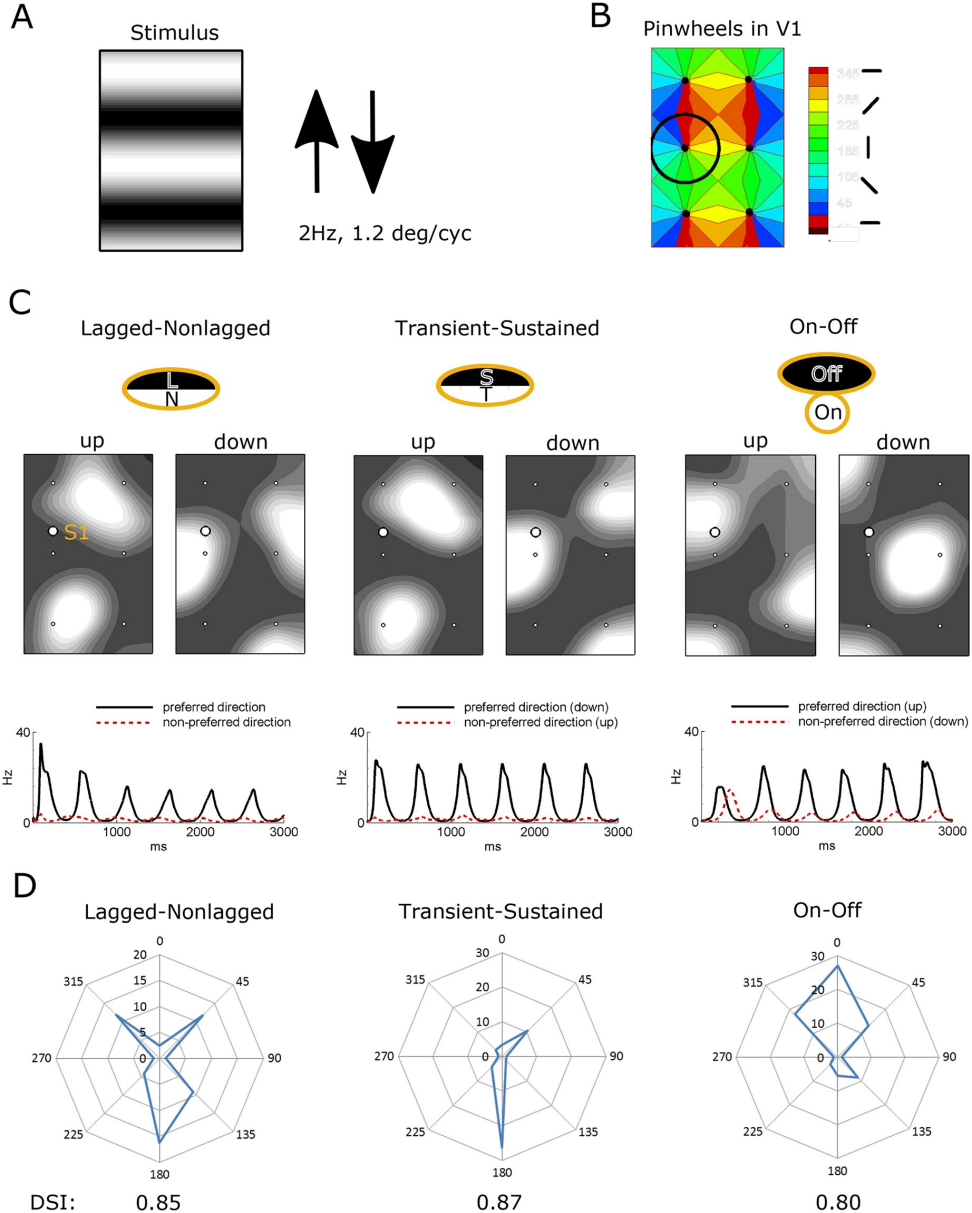
Responses to moving gratings reveal strong directional selectivity (DS) for three mechanisms. A. Stimulus within the domain that retinotopically projects onto the modelled cortical domain (B). B. The V1 area includes six orientation hypercolumns with pinwheels (dots). C. Three DS mechanisms with correspondent footprints (top row) give different patterns of the firing rate of excitatory neurons averaged over the initial 1600 ms time period (middle row). For all mechanisms, left panels are the responses to horizontal gratings moving up and right panels to gratings moving down. Neurons located under the white circle have the footprints shown above the activity patterns. The bottom row shows the firing rates of the excitatory neurons located within the white circles; the firing rate is much stronger for the downward than for the upward drift of the gratings. D. The tuning diagrams for the firing rate (in Hz) versus the stimulus direction (in degrees) at the site shown in C. The direction selectivity index (DSI) is given below.

We have examined the mechanism of DS by simulating responses to horizontal gratings of a spatial wavelength 1.2° moving up and down with the temporal frequency of 2 Hz (Fig 3A). Simulated cortical domain contained 6 orientation hypercolumns located, for simplicity, on a rectangular grid (Fig 3B). In response to the stimulus, the bright spots in the patterns of activity averaged over a large time period correspond with high rates of firing of the V1 excitatory neurons (Fig 3C). The spots appear in the columns that prefer an orientation similar to that of the stimulus. The patterns are not symmetrical with respect to the central vertical axis, which is due to DS, i.e., different direction preferences for neurons of the neighboring columns with the same orientation preferences belonging to different orientation hypercolumns. The peaks of E-cell activity are located in different hypercolumns depending on the direction of the grating movement. These simulated patterns are similar to those registered with optical imaging, such as those obtained in the cat visual cortex [6] (see their Fig 4A and 4B). The spots have a similar size and smooth shape and shift after a change in the stimulus direction.

**Fig 4:**
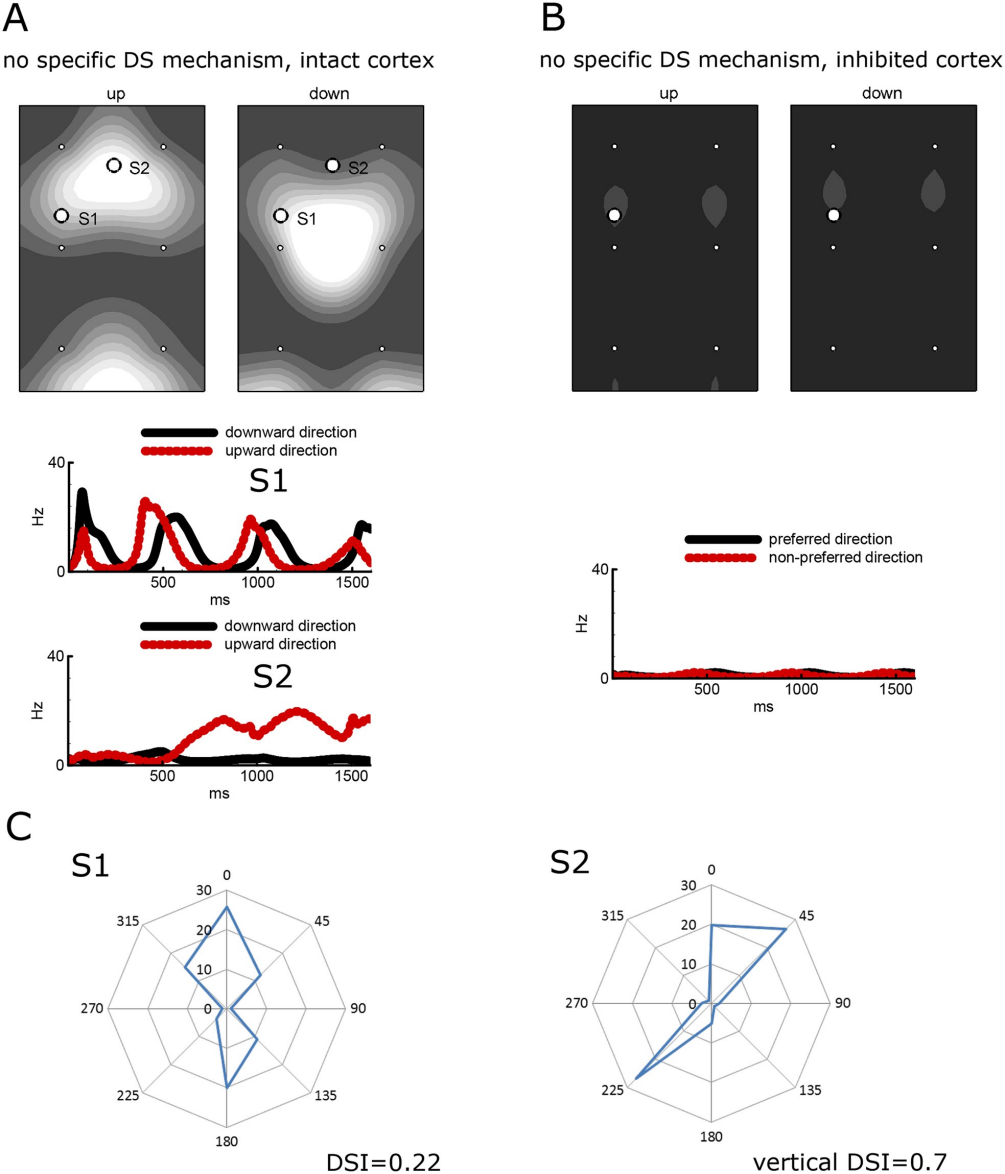
Responses to moving gratings reveal directional selectivity (DS) in the absence of any specific DS mechanism but not for the artificially inhibited cortex. A. Patterns of the firing rate of E-neurons averaged over the initial 1600 ms time period (top row) in response to the gratings moving up (left) and down (right) and correspondent traces at two sites S1 and S2 (middle and bottom rows). B. The same patterns (top row) and traces (bottom row) for the artificially inhibited cortex. C. The tuning diagrams for the firing rate at the sites S1 and S2 shown in A. The direction selectivity index (DSI) is given below.

The comparison of three DS mechanisms in Fig 3 shows that (i) DS is strong in all three cases (Fig 3C, **bottom**) and characterized with sharp tuning diagrams (Fig 3D), high values of the direction selectivity index (DSIs) for a representative site (Fig 3D) and considerable values of the DSIs averaged across the entire simulated cortical domain for the firing rate (0.29, 0.46 and 0.28 for the L-N, T-S, and On-Off models, respectively); (ii) DS maps are similar for L-N and T-S mechanisms; (iii) the DS map for the On-Off mechanism is different, though the number of spots is comparable; (iv) in all the cases, the direction response is not well-aligned with the orientation map shown in Fig 3B; (v) the T-S mechanism gives the most stable response on each cycle of the gratings; and (vi) the preferred direction (downward) in the L-N model corresponds with the direction from the lagged to nonlagged subfields of the footprint. The preferred direction in the T-S model corresponds to the direction from the sustained towards transient subfields.

### Pinwheel structure and intracortical interactions lead to DS without any subcortical bias

A question arises regarding to what extent DS may be caused by intracortical interactions unbiased with the structure of the footprints. To answer this question, we have considered a model with a footprint of the same elongation but without any discrimination of LGN cells within the footprint. Thus, the thalamo-cortical projections were set to be unbiased towards the stimulus direction. In this model, cortical neurons do not receive any precortical information about the stimulus orientation. At the same time, the pinwheel structure of the thalamic input provides an intracortical bias because a neuron may receive unequal signals from the neighboring neurons with similar and opposite preferences. The model shows that the response patterns are symmetrical (Fig 4A) according to the orientation map (Fig 3B), as expected; however, DS is still present, as seen from the tuning diagrams and DSIs given for two representative sites in Fig 4C. Though, DS is weaker (averaged over the entire domain DSI for the firing rate is 0.21), transient (Fig 4A, **bottom**), and the neurons preferring upward or downward directions are located in zones different from those for the three DS models discussed above. These facts along with the asymmetry of the patterns in Fig 3C lead to the conclusion that the direction maps obtained with the explicit DS mechanisms are determined by the precortical bias rather than the intracortical interactions. At the same time, the strength of DS may strongly depend on the intracortical interactions.

To reveal a role of intracortical connections (Fig 2A), the cortex was artificially inhibited. For that purpose we supply the inhibitory neurons with extra depolarizing current 100pA that mimicks the effect of photoactivation of cortical inhibitory interneurons expressing channelrhodopsin-2 [13]. It resulted in increased inhibition, which almost silenced the principle neurons.

In the case without any specific DS mechanism, the artificial inhibition of the cortex results in a significant reduction in the response (Fig 4B). The response is no longer selective to direction (Fig 4B, **bottom**); however, it is still weakly selective to orientation. Locations of the firing neurons (gray spots in Fig 4B, **top**) correspond to the horizontal orientation preference regions on the orientation map in Fig 3B. The dramatic decrease in the response in comparison with that for the intact cortex (Fig 3A) reveals a major role of the intracortical interactions in strengthening the tuning to feature detection.

### Contribution of cortical interactions is different for different DS mechanisms

**Fig 5:**
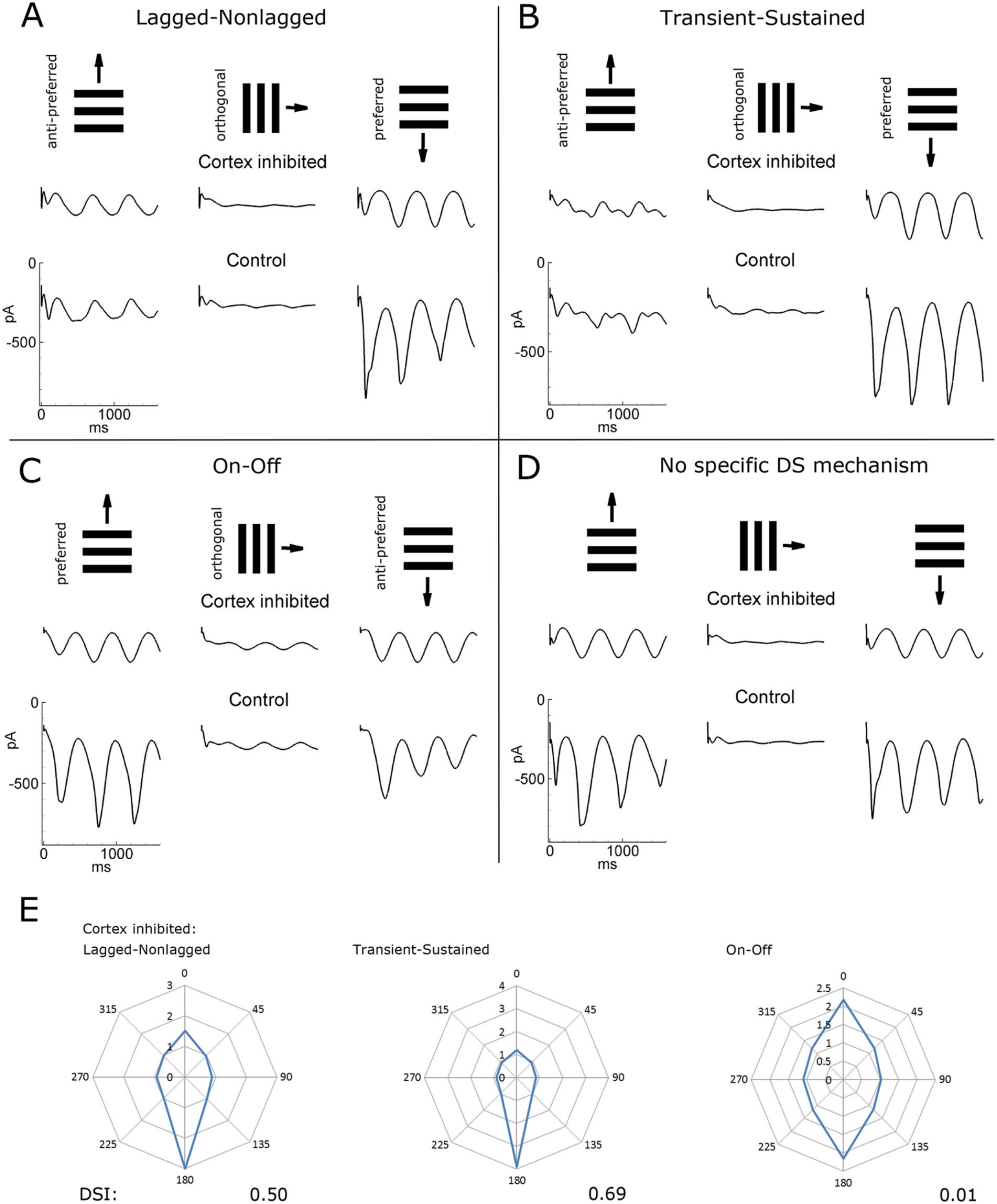
Inhibition of the cortex. Lagged-nonlagged (A), transient-sustained (B), and On-Off (C) models of DS as well as the case without any subcortical bias towards the direction (D). In each case, upper row–stimuli, middle and bottom rows–simulated V1 signals in the conditions of intact (bottom row) and the artificially inhibited cortex (middle row). The signals are from the representative neuron in the cortical site S1 marked by the white circle in Fig 3C. E. The tuning diagrams for the firing rate (in Hz) of the same neuron as in A-C in the case of inhibited cortex, to be compared with those for the intact cortex shown in Fig 3D. The direction selectivity index (DSI) is given below.

Different mechanisms of DS are compared in Fig 5. A significant transient but weak steady DS was observed with the On-Off mechanism (Fig 5C, intact cortex): the amplitude of current oscillations in the case of the preferred direction compared with the non-preferred one was significantly larger for the two initial cycles of the gratings, whereas it was only moderately increased for the later cycles. An even weaker DS was observed in the case without any directional mechanism (Fig 5D, intact cortex). Strong directionality can be observed in two cases—L-N and T-S mechanisms (Fig 5A and 5B, intact cortex)—in which the magnitude of the oscillations is significantly larger for the preferred direction. In all the cases, the responses to the orthogonal, horizontal gratings are smaller than for vertical gratings moving in both preferred and non-preferred directions, revealing strong orientation selectivity (see the tuning diagrams in Figs 3D and 4C).

Following Lien and Scanziani [13], we simulated responses of a V1 neuron to gratings moving in preferred and non-preferred directions under the conditions of an artificially inhibited cortex for each of the three DS mechanisms (Fig 5). The amplitudes of the firing rates are much smaller with the inhibited cortex compared to the intact cortex. The effect of cortical inhibition is different in the three models. A weak direction selectivity could still be observed for the L-N and T-S mechanisms (Fig 5A and 5B, cortex inhibited; Fig 5E), but no difference in the response to the preferred and non-preferred stimuli was evident for the On-Off mechanism (Fig 5C, cortex inhibited; Fig 5E), such as in the case without a DS mechanism (Fig 5D, cortex inhibited). For the On-Off model, a weak temporally modulated response was observed even when the gratings moved in the orthogonal direction, which was not the case for the other models. The direction tuning diagrams for the inhibited cortex (Fig 5E) demonstrate weaker tuning than those for the intact cortex (Figs 3D and 4C). The latter ones are more sharp because of intracortical shunting inhibition [43].

#### Vertical DSI patterns

**Fig 6:**
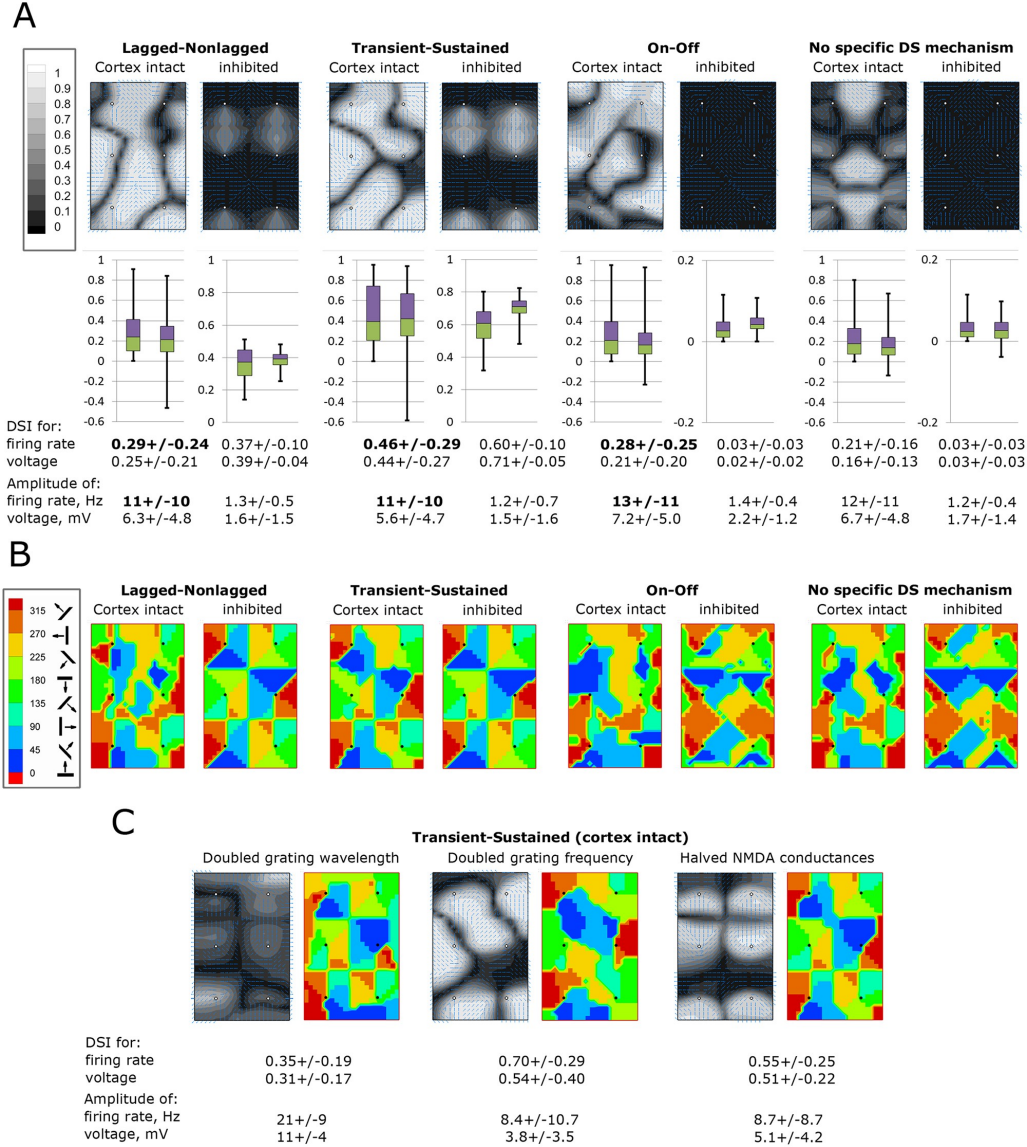
DSI and the direction maps. A. The index of preference of the vertical direction distributed in the cortical domain, calculated for the excitatory neuron firing rate obtained in 8 models presented in Fig 5A–5D. Distribution of direction selectivity index (DSI) values is characterized by the box plots. Spatially averaged values of the DSI calculated for locally preferred directions are given at the bottom (mean+/-standard deviation), for both the firing rate and the membrane potential of the excitatory population, as well as the averaged across time and space firing rate and voltage modulation. Note that the DSI for voltage can be negative, when calculated for the preferred direction pre-determined with the firing rate. B. The preferred direction maps. Pinwheel centers are marked by dots. C. The vertical DSI distribution and the preferred direction maps for the T-S model with modified parameters: (i) doubled wavelength (2.4 deg) of the stimulating gratings; (ii) doubled frequency (4 Hz); (iii) halved NMDA conductances.

In the case of the intact cortex, the distribution in the cortical space of the DSI is calculated for the vertical direction, which reveals complex, asymmetrical patterns for the L-N, T-S, and On-Off models (Fig 6A, left panels of each of the left three pairs) that do not correspond to the thalamic input pattern structured by the orientation map of the thalamic signal, shown in Fig 3B. In contrast, the inhibition of intracortical interactions leads to either symmetrical distributions of the vertical DSI (Fig 6A, right panels for L-N and T-S models) or disappearance of the selectivity (Fig 6A, right panels for the On-Off model).

#### Mean DSIs

In the case of the intact cortex, mean DSIs for the firing rate are high for the L-N, T-S, and On-Off models (0.29, 0.46, and 0.28, respectively) and are comparable with those observed in animals [44,45]. The inhibition of the cortex results in an expected crucial reduction of the mean firing rate (from 11-13Hz to about 1Hz). DSIs increase in the L-N and T-S models (0.37 and 0.60, respectively), which shows that intracortical tuning has a “contaminating” effect of as a price for amplification. DS vanishes in the On-Off model, as expected and explained at the beginning of the Results section. In the case of the intact cortex, the DSI values based on the firing rates are greater than those for the voltage (Fig 6A, compare the numbers in the bottom two lines), which reveals the threshold nonlinearity as in experiments [13,44]. The effect is opposite for the inhibited cortex where most voltage modulations are subcortically evoked, subthreshold, and thus do not modulate the firing rate.

#### Direction maps

Complex patterns are also observed in the preferred direction maps for the intact cortex (Fig 6B). The patterns for the inhibited cortex are more regular, being structured by the thalamic input patterns. (The patterns for the inhibited cortex in the On-Off model and the model without any specific DS mechanism are insignificant because of negligible DSIs.) That the preferred directions of the spike response differ from those of the thalamic input is consistent with experimental data [13]. This difference is revealed to be due to cortex inhibition, which shows the contribution of intracortical interactions.

*The model without any specific DS mechanism* has a symmetrical vertical DSI pattern in case with an intact cortex (Fig 6A, left panel in the right pair), in contrast to the other models. The DS is considerable (mean DSI 0.21). The direction map (Fig 6B, left panel in the right pair) is determined mostly by the orientation-tuned thalamo-cortical projections and intracortical interactions. Because of the model’s construction, this map is not affected by any specific tuning of input or intracortical connections to the directions of stimuli. That is whythe similarity between this map and those of other models with an intact cortex (Fig 6B, left panels of each of the left three pairs) reflects a contaminating contribution of the cortex on the direction tuning of neurons, different from the direction tuning caused by the thalamic input. The direction map of the T-S model is the least affected by intracortical interactions.

#### Variation of parameters

We varied some parameters of the stimulating gratings in the T-S model (Fig 6C) and compared the simulated results with the control (Fig 6A and 6B, third panels). The comparison show that DS is stronger for faster and finer stimuli (DSIs for the firing rate were 0.29, 0.46, and 0.70 for 1, 2, and 4Hz, respectively; 0.64, 0.46, and 0.35 for 0.9, 1.2, and 2.4 degrees, respectively). This is in contrast to the response amplitudes, which are greater for bigger and slower visual patterns (maximum firing rates were 12, 11, and 8.4 for 1, 2, and 4Hz; 5.4, 11, and 21 for 0.9, 1.2, and 2.4 degrees, respectively). The blockage of NMDA receptors is known to reduce visual responses without significantly changing the degree of DS [46–48]. In our simulation, halving the NMDA conductance (Fig 6C, right panels) consistently increased the mean DSI (from 0.46 to 0.55) with a reduced firing rate (from 11 to 9Hz) and produced avertical DSI pattern much closer to the one for the inhibited cortex (compare with Fig 6A) and the DS map that is an intermediary between the maps for the intact and inhibited cortex.

### Synaptic activity underlying DS

**Fig 7:**
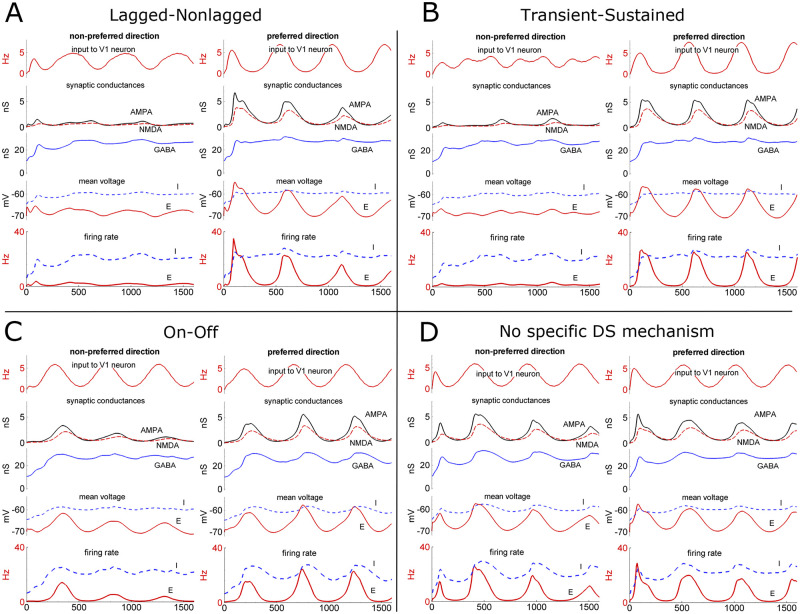
Intracortical activity. Signals from one site of V1 (S1, circle in Fig 3C) in response to gratings moving in non-preferred (left) and preferred (right) directions in the lagged-nonlagged (A), transient-sustained (B), and On-Off (C) models of DS, as well as in the case with no specific DS mechanism (D). Simulations as in Fig 4. Top to bottom: the presynaptic firing rate received by V1 excitatory neurons (magenta); dimensionless excitatory (AMPA [black] and NMDA [red]) and inhibitory GABA (blue) synaptic conductances at the intracortical recurrent connections; the mean membrane potentials of the E- (red) and I- (blue) populations; the E- (red) and I- (blue) population firing rates. The NMDA conductance here does not consider the factor of the voltage-dependent magnesium block. The AMPA conductance was multiplied by 10 to be compared with the GABA and NMDA components.

Synaptic mechanisms of DS in different models are illustrated in Fig 7. For the location S1 marked by a circle in Fig 3C, the LGN input, synaptic conductances, mean voltage, and firing rate are shown in Fig 7 for all the models: L-N (Fig 7A), T-S (Fig 7B), On-Off (Fig 7C) and with no specific DS mechanism (Fig 7D). These simulated signals are similar to experimental data, such as those from [43,44] and [49] (their Fig 5). As expected, the shape of the membrane potential that is traced in response to the preferred stimulus is close to sinusoidal, with maxima and minima corresponding to the oscillations of the gratings and without extra peculiarities. The mean peak amplitude of voltage modulation was about 10mV in the L-N, T-S and On-Off models, which is within the range of the experimental values (mean depolarization 9.5mV and hyperpolarization 3.7mV in [43], voltage modulation about 22 mV for the representative cells in [44] (their Fig 2A), 10mV in [50] and 8mV in [49]). In response to the non-preferred stimulus, this measure is about 2mV in our models, 12mV in [44], 5mV in [50], and 2mV in [49]. Our peak value for the firing rate of excitatory neurons is about 20Hz, which does not exceed the experimental values (80Hz in [44], 40Hz in [43], and 50Hz in [13]). The firing rate bumps are more narrow than those for voltage, similar to those of experiments [43,44]. The input and synaptic conductances are within the range of experimental values. The input conductance of excitatory neurons at the resting state in the model is about 18nS, which is comparable to the mean value of 16nS in the experiment [49]. The total synaptic conductance reaches 30nS in our models. After subtracting the total synaptic conductance in the state of the gray screen stimulation (9nS), the synaptic conductance modulation (21nS) is comparable to the mean experimental value (16nS) from [49].

As shown in Fig 7A–7C, in all three DS models, the oscillations of the thalamic input to V1 in the case of non-preferred direction are small. The thalamic input oscillations in the case of the preferred direction are similar in all the models. The strongest tuning of the input to the cortex was observed in simulations with the T-S model. The total set of input signals for any neuron consists of NMDA, AMPA, and GABA synaptic conductances that include contributions of both thalamocortical and intracortical connections. NMDA conductance is the largest component in the responses of all the models; however, note that this component is voltage-dependent due to magnesium blockage, and thus to exclude an effect of voltage oscillations, the NMDA-signal is plotted with the voltage-dependency factor fixed to 1, as if for zero-magnesium conditions. Therefore, its values are not to be directly compared with the AMPA and GABA conductances. In any case, all three synaptic components react to the change of stimulus direction. Oscillations of glutamatergic components are the most distinct features in the comparison between the responses to preferred and non-preferred stimuli. It is the main source for the voltage and firing rate modulations that produce DS. Comparing all models, the oscillations of the NMDA-component in the case of the preferred stimulus are the largest and most stable for the T-S mechanism.

In contrast to NMDA and AMPA, the GABA-component was rather constant over time (Fig 7). Minor GABA- and larger NMDA- and AMPA-oscillations were in-phase. Both mean voltage and firing rate oscillate during stimulus drift in all the models. In contrast to the T-S model, the oscillations in the L-N and On-Off models were larger for the first and second cycles of the gratings and then stabilized (see Fig 3C and compare Fig 7B to Fig 7A and 7C). A strong two- or three-fold decrement in both mean voltage and firing rate was observed up to the third wave, but thereafter, the oscillations were stable. The oscillations of firing rate are stable on larger time scale (Fig 3C). Therefore, the simulations indicate that the T-S model as the most reliable for DS.

In the case with no specific DS mechanism (Fig 7D), for the particular site of the cortex (S1), shown in Fig 4A, synaptic conductance modulation is similar for both directions of the stimulus according to the tuning diagram (Fig 4C). The magnitude of the conductance modulations is comparable to those for the L-N and On-Off models, pointing again to the dominant role of intracortical activity evoked not by directional but by orientation-tuned thalamic input.

**Fig 8:**
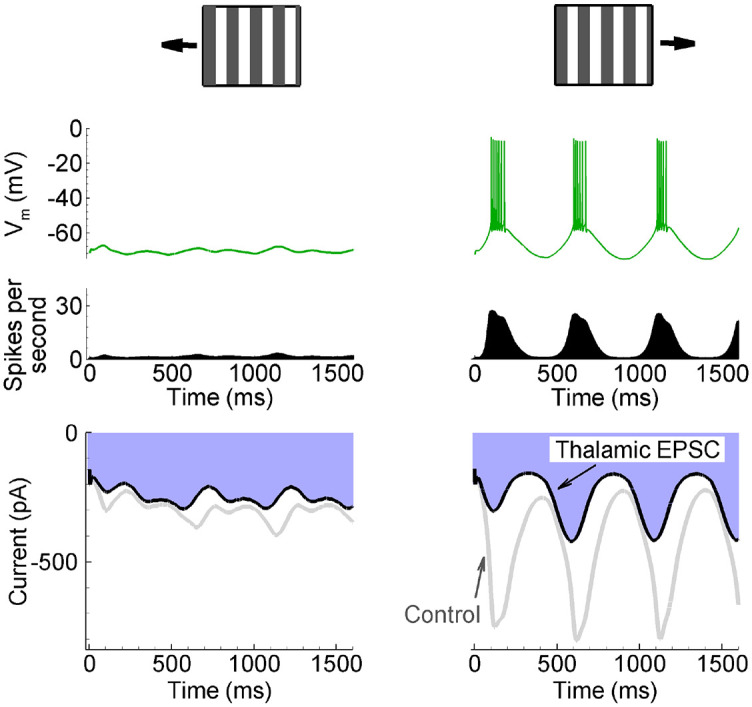
Amplitude modulation of thalamic excitation is direction selective in the T-S model. Response to moving gratings of the non-preferred (left) and preferred (right) directions in in the transient-sustained model of DS as in the experiment by Lien and Scanziani [13] (see their Fig 1c and 1e). Top to bottom: the membrane potential of a representative excitatory neuron (green); the population firing rate (black); the current as though it was recorded in the voltage–clamp at the holding voltage of -70mV in the cases of an intact (Control, grey) and an inhibited cortex (Thalamic EPSC, black). The signals are from the representative neuron in the cortical site S1 marked by the white circle in Fig 3C.

The CBRD-model allows for the reconstruction of a representative neuron’s behavior with input variables, such as synaptic conductance, known from the population activity. We simulated a representative neuron of the E-population in the T-S model to compare it with experimental data from [13] (see their Fig 1c and 1e), where the T-S mechanism has been highlighted. The representative E-neuron generates spikes only during the presentation of the preferred direction (Fig 8, **top right**) whereas, in the case of the non-preferred direction, only subthreshold depolarization was observed (Fig 8, **top left**). These voltage traces recorded in response to moving gratings are consistent with those observed in experiments *in vivo* when the shape and amplitude of voltage oscillations are compared [13] (see their Fig 1 and present Fig 8), [49,51]. In the null direction, the voltage and current modulation are significantly larger and spikes are much more numerous. As explained in [13], the temporal modulation of the excitatory postsynaptic current (EPSC) provided by the thalamic input is different for the preferred and non-preferred stimuli, whereas the integrated charge (Q) is maintained (compare the fulfilled areas in Figure 1 from [13] and Fig 8, **bottom right and left**). Whereas the charges in our intact cortex simulations were different for the preferred, orthogonal, and opposite directions (470, 216, and 259pC, respectively), the charge was the same for the thalamic EPSC evaluated with cortex inhibition (205, 196, and 197pC). The maintenance of the total input charge in the experiment and in our simulations verifies that the transient and sustained neurons are independently active and are not biased toward the stimulus direction. Indeed, the stimulus direction does not affect the footprint of a V1 neuron (Fig 3C **top middle**) and changes only the timing of the signals from the T-S neurons. This is why the averaged in time thalamic input is independent of the stimulus direction. In contrast, the temporal modulation of the current is determined by the correlation between the firing rates of those T- and S-neurons that contribute to the footprint; it therefore depends upon the stimulus direction.

Thalamic contribution was estimated in [41] as the ratio of the charge due to the thalamic EPSC to the charge due to the EPSC in the intact cortex, *Q*_*control*_/*Q*_*inhibited*_, which was found to be 0.36 for stimulation with gratings of preferred directions. In our simulations, the values of this ratio for responses of the representative neurons located in S1 in the L-N, T-S, and On-Off models were 0.46, 0.43, and 0.47, respectively (Fig 5A–5C), which are comparable with the experimental values. The mean values of the ratio *Q*_*control*_/*Q*_*inhibited*_ for all neurons preferring the same stimulus direction in the L-N, T-S, and On-Off models were 0.42 ± 0.05, 0.41 ± 0.07, and 0.50 ± 0.10, respectively. Note that the ratio values for S1 match the mean values.

### Identification of a prevailing DS mechanism

**Fig 9:**
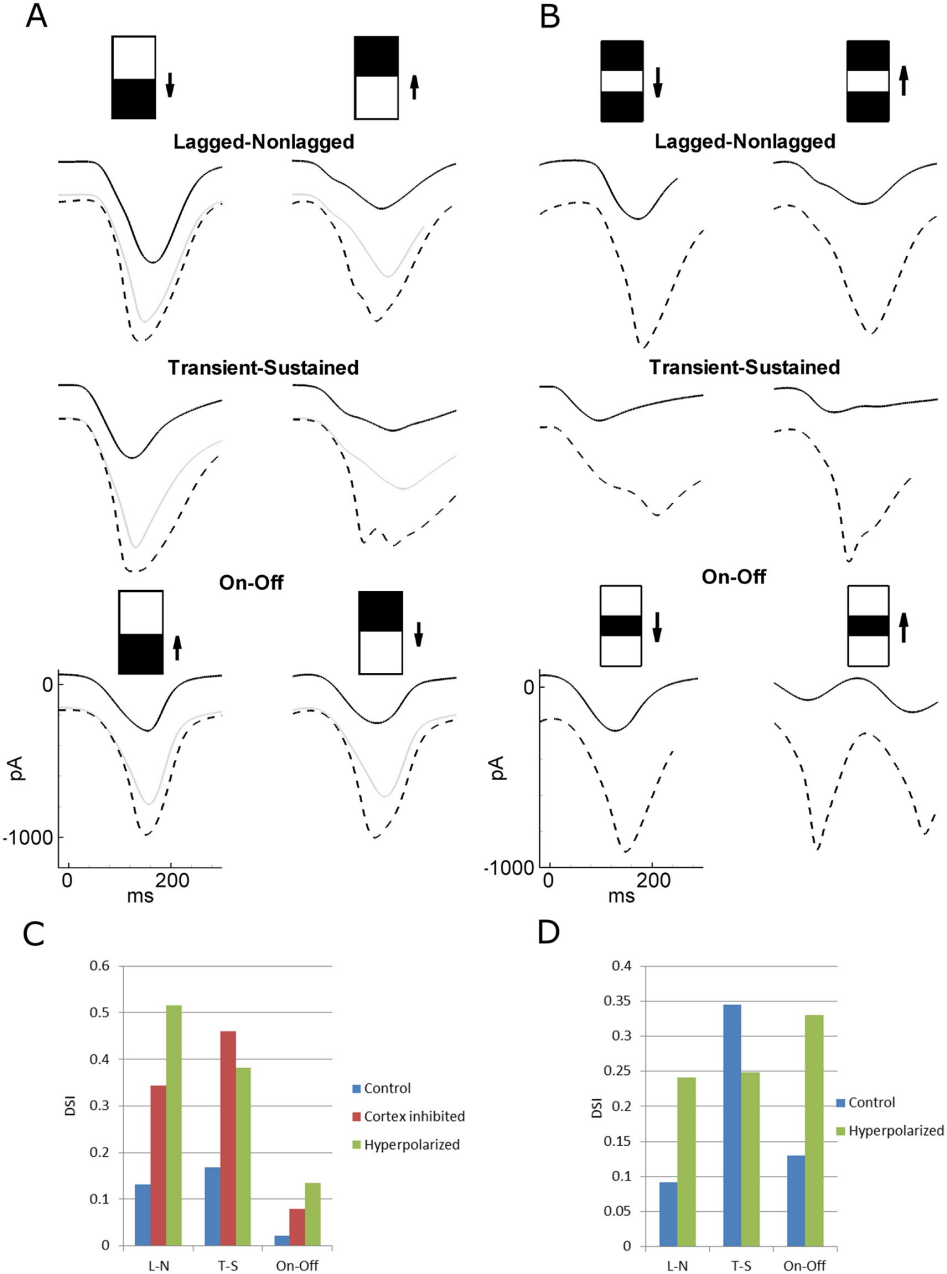
Stimulation with a white-black edge and a bar. A. Responses to black-white and white-black edges moving in opposite directions (left versus right plots) in the lagged-nonlagged, transient-sustained, and On-Off models under the conditions of the intact cortex (dashed line), or inhibited cortex by either depolarization of interneurons by an injected current of 100pA (gray line) or hyperpolarization of the excitatory neurons by an injected current of -200pA (black solid line). DS is evident in the L-N and T-S models, not in the On-Off model. B. Responses to a bar. Legend as in A. C and D. DSI for the simulations in A and B. In the On-Off model with hyperpolarization, DS is evident with a bar-stimulus, not an edge. The signals are from the representative neuron in the cortical site S1 marked by the white circle in Fig 3C.

The most important question related to DS is likely how a prevailing DS mechanism can be identified. Though responses to drifting gratings have distinct characteristics in different DS models, they are not necessarily robust criteria. For instance, as seen from Fig 7C, the tuning of the thalamic input in V1 is very weak for the On-Off model; however, a moderate directionality was still observed (Figs 3C, 5C and 7C). This is mostly due to the periodicity of the gratings used as a stimulus. As noted at the beginning of the Results section, as weak DS is expected in the On-Off model for stimulation with gratings or for stimuli that excite only the On or Off subzone of the footprint. In contrast, a non-periodic stimulus that excites both subzones is expected to reveal DS in the model. To determine the difference between the On-Off and other DS mechanisms, we propose the application of an experimental protocol with a non-periodic stimulus containing only a moving edge between white and black domains of the screen (i.e. an initially black or white screen changing to white or black, respectively, with a moving edge between the domains of different light intensities [Fig 9]). However, to avoid contamination due to intracortical interactions, the experiment also requires inhibition of the cortex. The simulations showed that an inhibition in the form of the opto-driven excitation of interneurons, as applied in [13], is not sufficient to prevent contamination (Fig 9, gray lines). The principal neurons of the cortex must be silent. For this purpose, a hyperpolarization of neurons must be provided. During experiments, it can be done, presumably by means of halorhodopsin. In simulations, we modelled such hyperpolarization by an additive negative current (-200pA) injected in E-neurons. Under these conditions, the responses of the On-Off model are qualitatively different from those of the L-N and T-S models (Fig 9A and 9C), showing an absence of DS only in the On-Off model. It is in contrast to the response to a bar (Fig 9B and 9D), which is directionally selective even for the On-Off model with hyperpolarized principle neurons, i.e. with for the thalamic input in this model. This qualitative difference between the responses to an edge and a bar is observed only for the On-Off model. Hence, a similar experimental protocol might help establish the contribution of the On-Off mechanism to DS.

## Discussion

In the present modeling study, a previously elaborated model of the neuronal population activity in the neocortex [34], which describes an orientational selectivity, has been adapted for DS as well.

### Modeling approach advantages

Responses to visual stimuli, such as gratings, are determined by network activity, to which neuronal populations rather than single neurons substantially contribute. At the same time, this activity is often studied by means of intracellular electrode registrations. Thus, an ideal approach for mathematical modeling should describe the network activity and the activity of recorded representative single neurons, whereas a direct description of all neurons of the network might be considered redundant. This detailed but population-type approach of modeling suits these idealized requirements and contrasts with the other comprehensive models, such as the most prominent ones [35,52] in which the V1 network is directly simulated with a large number of individual neurons. As an advantage, our approach provides an even wider set of experiments captured by the model if those described in our previous paper on orientation tuning and a comparison between *in vitro* and *in vivo* experiments are included [34].

Contrary to the detection of orientation based on mainly spatial peculiarities of the receptive fields, the detection of stimulus direction also requires temporal peculiarities of the responses. Thus, a temporal shift between the inputs should be taken into account. A strong DS of the V1 neurons has been observed in the three cases of the integrated thalamic input received from neurons with: (i) lagged and non-lagged, (ii) transient and sustained, and (iii) On and Off receptive fields.

### Differences between the three models

Quite similar levels of DS in the cases of L-N and T-S thalamic inputs (see Figs 3C, 5A and 5B) were observed, and a weaker but comparable DS in the case of On-Off inputs was observed. Both the mean voltage and the firing rate oscillate during the stimulus motion. These oscillations were the most stable in the T-S model (Fig 7B). In the On-Off (Fig 7A) and L-N (Fig 7C) models, DS was more evident for the first and second cycles of the gratings, whereas a strong three-fold decrement in both the mean voltage and the firing rate was observed for the third and following cycles. These facts justify favoring the T-S model; however, the transient DS provided by the other models can be also considered functionally sufficient for the secondary visual processing that receives information from the primary visual cortex fragmentarily, separated on the time intervals of eye fixations between saccades. It is interesting that non-periodic stimuli (single pair of black and white stripes, not gratings) result in the most prominent difference between the models (Fig 9). Another stimuli, such as moving dots [53], could also potentially be helpful to distinguish between the models; however, our simulations were limited by computational resources and thus considered only a small area of the cortex, so widespread stimuli could not be processed. Thus, this issue is a matter of future investigations.

The weakest directionality was obtained in the On-Off model. The On-Off division of the visual cells is the first within the visual processing pathway [20,54] and is believed to underlie a DS at the retinal level in lagomorphs and rodents [15,55]. This is not the case for animals with more developed vision, such as carnivores and primates, where the predominant role in DS belongs to higher structures, meaning the visual thalamus and the cortex. Accordingly, the On-Off thalamic mechanisms in these animals (like in our mathematical model) could play a secondary role in the cortical DS. This aspect aligns with the finding that the blockade of an On pathway has no discernible effect on orientation and direction specificities in the cortex of rabbits [56].

For the T-S mechanisms, the question about an origin of DS is related to a separation of the visual system into two main processing streams, so-called X and Y channels, provided by corresponding retinal ganglion cells and thalamic cells. One specific characteristic of the Y and X cells is the duration of their activity after a stimulus onset: fast for the “transient” cells (Y cells) and prolonged for the “sustained” cells (X cells) [16,17,57]. For many years, it has been supposed that only Y cells are responsible for motion processing [58], but D. Mastronarde, A. Humphrey, and A. Saul [19,20] suggested that this is not the case. Instead, the temporal shift between the responses of lagged and non-lagged counterparts irrespective of the X and Y types underlies DS in the visual cortex. The prominent DS in both the L-N and the T-S models is in favor of this suggestion.

### Sources of tuning

Tuning to stimulus direction is provided by the cumulative action of the following factors: (i) the projections of LGN to V1, described in Section 2.3 (Fig 1), (ii) the threshold rectification in V1 neurons, and (iii) the recurrent intracortical interactions. Indeed, the LGN input to V1 excitatory neurons is tuned. This tuning is more evident in the T-S model (compare left and right traces in Fig 7A) and is reflected in EPSC recorded in the inhibited cortex in L-N and T-S models (Fig 5A and 5B); it is less evident in the On-Off model (compare left and right traces in Fig 7B) and in EPSC recorded in the inhibited cortex (Fig 5C). Trivially, it is absent in the model without any DS mechanism (Fig 5D). The second factor results in stronger tuning of the mean membrane voltage of E-neurons than of I-neurons (Fig 7), which is due to the effect of recurrent synaptic inputs. The inhibitory interneuronal activity reflected by the GABAergic conductance on the excitatory neurons contribute to the tuning of voltage with depolarization relative to the spike threshold. The threshold rectification produces the firing rate and thus sharpens voltage tuning. Finally, the firing rate determines the probability for a single neuron of a population to generate spikes, as shown in Fig 8. As a result, the preferred direction is determined by the cumulative action of those factors, not simply by the preferred orientation of the thalamic input, which is consistent with experimental findings [13].

### Synaptic conductances underlying cortical tuning

Our simulated conductances are not to be directly compared with experiment-based estimations. In our simulations, the GABA-component during gratings was rather constant over time; minor GABA- and larger NMDA- and AMPA-oscillations were in-phase (Fig 7). On the contrary, known experiment-based estimates of synaptic conductances [44,49,51,59] report that the excitatory and inhibitory conductances oscillate in antiphase. It should be noted that our simulated variables should not be directly compared with those estimates. Rather than three separate (AMPA, NMDA, and GABA) components, the mentioned experimental studies reported estimates of two conductances: excitatory and inhibitory. This method, due to not taking into account the voltage-dependence of NMDA receptor magnesium block, overestimates oscillations of the inhibitory conductance and gives in-phase modulations of the excitatory and inhibitory signals. This limitation of the estimation procedure has been recently revealed [60]. Our current simulations confirm that it is not an antiphase interaction of inhibition and excitation but rather oscillations of the excitatory synaptic drive that underly the temporal modulations of the firing rate response, which contradicts to some earlier models based on the mentioned estimations [61,62] but consistent with more recent work [35] and with the study emphasizing the role of NMDA receptors in cortical temporal tuning [63]. Similar data on the DS of the spike responses and membrane depolarization responses and excitatory and inhibitory interactions underlying DS were obtained using *in vivo* whole-cell voltage-clamp recording; it was shown that (1) both spike responses and membrane depolarization are sensitive to the stimulus direction, and (2) the bias of the inhibitory responses was much weaker than that of excitation or even not observed [64].

### Cortical inhibition and directionality

In L-N and T-S models, the artificial inhibition of the primary visual cortex decreased the directionality level but did not totally disrupt it; this is mostly related to the T-S model. This might mean that cortical interneuronal communication enhances directionality. It is well-known that cortical neurons with a preference for a particular direction are grouped into directional columns [6,65]. Many studies documented patchy patterns of cortical horizontal interconnections that have approximately the same center-to-center distance, such as between functional columns [66–69]. In accordance with the wiring economy thesis [70], this connectional pattern, such as cortical columns *per se*, can be a result of an attempt to minimize the wiring cost for the intracortical connectivity. In this case, a reduction in directionality due to cortical inhibition is expected. Moreover, the major role in strengthening directional tuning belongs to intracortical interactions (Fig 4). This could mean that DS may be caused by the inherent intracortical interactions unbiased by thalamic footprints.

Our modeling helps to estimate the role of pure intracortical interactions, which in the presence of a pinwheel structure of the cortex could lead to a DS that is independent of any thalamocortical input bias to stimulus direction. In fact, we have observed DS in this model, which is much weaker than that in the L-N, T-S, and On-Off models. Naturally, DS disappears after silencing the cortex. The effects of this mechanism cannot be completely excluded for intact cortex functioning. Moreover, these intracortical interactions within the orientation map are critical in allowing DS to emerge in the On-Off mechanism.

### Development of the model

Because the detailed simulation presented here is consistent with the experimental data, in particular with those reported in A. Lien and M. Scanziani’s paper [13], our CBRD-model is recommended for a prognostic modelling of hypothetic experimental conditions, such as the activation/blockage of a particular thalamic input, changing cortical connections, taking into account particular types of GABAergic neurons, and alterations of neuromodulators and cellular messengers. In a future work, it is also worthwhile to compare model predictions with *in vivo* data on wild-type and knock-out animals. For example, bursting properties of the cortical pyramidal neurons are determined by the hyperpolarization-activated cationic current [71]. The hyperpolarization-activated cyclic nucleotide-gated channels (HCN) underlie this current; within the CNS, these currents were first described in the retina [72]. In HCN knock-out animals, a special compensatory upregulation of the tonic GABA-A currents was revealed [73,74]. In the retina, it was observed that directionality is dependent on the HCN channels [75]. These conditions can be simulated with the proposed mathematical model.

The synaptic connections in our model were chosen without any optimization tuning. A task-aimed parameter tuning is expected to result in better visual processing performance, as in the model for motion processing in the MST area that uses an unsupervised optimization technique based on multi-cause and principle component analysis [76] or the model that uses Hebbian-like learning to tune the V1 model [35].

The model does not distinguish between different layers of the cortex, which is a reduction of our former model, in which layers 4 and 2/3 were explicitly present [34]. This reduction was made for simplicity. At the same time, we suppose that the transitions from layer to layer provide sharper tuning without a strengthening of recurrent excitatory connections [77]. The effects of multiple layers can be considered in future research.

### Model limitations

The major limitation of this model is the structuring of the visual cortex by functional columns. The population approach is efficient in this case although its usage is not as reasonable in case of salt-and-pepper distribution of orientationally and directionally selective neurons. In the case of the cortex structured by pinwheels, the direction and orientation are implicitly coded by x-y coordinates and do not contribute to the set of independent variables. In contrast, in the salt-and-pepper case, the functional variables would increase the dimensionality of the mathematical problem.

Another model limitation is related to characteristics of thalamic cells as sources of input to cortical cells. The main sources of the geniculate input to the area V1 are layers A and A1 (in carnivores) or magno- and parvocellular layers (in primates). As is known, layers A and A1 are different, and several different features in addition to the ocular dominance of their cells were observed. For example, electrophysiological studies report that On-cells are predominant at the top of the A-layers and Off-cells at the bottom, whereas both types are balanced at the centers of the A-layers. The steepest gradients and maximum differences in the proportions of On- and Off-cells have been obtained in layer A [78]. For the transient and sustained neurons associated with Y and X cells [16,79,80], in layer A, Y cells are concentrated near the interlaminar borders and X cells in the centers of the layers, but these patterns are less obvious for layer A1 [78,81]. Our own data related to the postnatal development of a cat’s LGN provides additional evidence of the different dynamics of the maturation of layers A and A1 [82]. These data together allow for assuming there are unequal contributions of the geniculate layers A and A1 to cortical DS. This aspect has not been taken into account in our model because only functional roles and not spatial placements of the thalamic neurons are important in the considered DS mechanisms.

Also, the model does not take into account a heterogeneity of either exciting or inhibitory cortical neuron populations. It is well-known that cortical inhibitory networks consist of multiple types of inhibitory neurons [83]; their role in cortical directionality is unclear. To clarify this aspect, the mathematical model can be further developed to describe the behavior of different neuronal types separately.

### Main predictions to be verified in experiments

The model predicts that the thalamic input biased toward DS and the intracortical interactions not specifically tuned to DS can together provide an efficient motion detection by cortical neurons in the presence of an orientation map structure. Hypothetically, this statement could be proven or disapproven in experimental settings in which the synaptic plasticity can be switched off specifically for intracortical connections, thus leaving the thalamic projections intact during the development of an animal, knowing that DS appears at a certain age [45]. A lack of strong DS in such treated adult animals would disprove the main model prediction. On the other hand, it is straightforward for the model to account for intracortical connections tuned to the direction preferences of the connected neurons, which is expected to increase DS. Patchy connections primarily between neurons with similar orientation preferences and, presumably, direction preferences are neglected in the model. These connections could drastically change DS, so this aspect should be verified in experiments.

The separation of the thalamic footprints’ subfields (Fig 1B) is another assumption of the model, similar to the assumptions made in alternative modeling studies [35,36]. Disapproval of these assumptions would ruin the mentioned models.

Another model prediction mentioned above concerns the synaptic conductances underlying cortical tuning. This prediction can be tested with experimental reconsideration of synaptic estimations in animals with pinwheels in vivo during stimulations with gratings, as proposed in [60] or with alternative methods. Evidence of considerable oscillations of GABA-mediated conductance in antiphase with glutamatergic conductances would demonstrate that the present intracortical tuning of the model is incorrect.

The model emphasizes the importance of strong recurrent excitatory connections and associates this tuning with a prolonged response to short activation. If this association is incorrect, the model should be re-tuned in favor of DS specificity in the connections.

In conclusion, the proposed biophysical model realistically reproduces the activity of the visual cortex, reveals the difference between the DS mechanisms, and helps to elaborate an experimental protocol that would reveal a prevailing mechanism of DS. These findings along with the mentioned previous modeling of orientation selectivity are a step towards a comprehensive biophysical modeling of the primary visual system in the frameworks of the population rate coding concept.

## Methods

### Model of stimulation

Model stimuli are black-and-white sine-wave gratings of various orientations (from 0° to 180°) and the spatial frequency 0.83 cycles per degree moving in two opposite directions orthogonal to the orientation at a 2 Hz rate. Stimuli parameters were chosen close to those found optimal in experiments [84].

The gratings are moving relative to the center of the screen (*x*_*c*_, *y*_*c*_) with some orientation *θ*_0_, wavelength *λ*, frequency *ν*, amplitude of luminance modulation *S*_*A*_ and background luminance *B*:

$$S(x,y,t)=B+S_{A}\;\text{cos}(2\pi (\sqrt{{\left(x-x_{c}\right)}^{2}+{\left(y-y_{c}\right)}^{2}}\;\text{cos}[arctg(\left(x-x_{c})/(y-y_{c}\right))+\theta_{0}]/\lambda -\nu \;t))\;,$$ (1)

A basic set of the parameter values was as follows: *θ*_0_ = 0, *λ* = 1°, *ν* = 2*Hz*, *B* = 50, *S*_*A*_ = 40.

### Model of LGN, and input to V1

We distinguish different types of LGN neurons that innervate V1 neurons, lagged and non-lagged, transient and sustained, and On- and Off-cells, whose activity is characterized by the firing rates $L_{LGN}^{L}(x,y,t)$, *L*_*LGN*_(*x*, *y*, *t*), $L_{LGN}^{T}(x,y,t)$, $L_{LGN}^{S}(x,y,t)$, $L_{LGN}^{On}(x,y,t)$ and $L_{LGN}^{Off}(x,y,t)$, respectively. We assume that the non-lagged, transient and On-populations are identical, i.e. $L_{LGN}^{T}(x,y,t)=L_{LGN}^{On}(x,y,t)=L_{LGN}^{}(x,y,t)$. At any given time moment, the firing rate of an LGN neuron is calculated as a convolution of the stimulus with a receptive field (RF). The firing rate *L*_*LGN*_(*x*, *y*, *t*) at any given time moment *t* is a rectified convolution of the stimulus *S*(*x*, *y*, *t*) distributed across the retina with a spatial-temporal receptive field (RF) *D*(*x*, *y*, *t*) [85]. The firing rate is calculated as follows:

$${\tilde{L}}_{LGN}(x,y,t)=\int_{0}^{t}d\tau \;\iint dx\prime \;dy\prime \;D(x\prime ,y\prime ,\tau )\;S(x\prime ,y\prime ,t-\tau )$$ (2)

$$L_{LGN}(x,y,t)={\left[{\tilde{L}}_{LGN}(x,y,t)\right]}_{+},$$ (3)

where the receptive field *D*(*x*, *y*, *t*)is approximated as the difference of central (excitation for On-neurons) *D*^*cen*^(*x*, *y*, *t*) and surround (inhibition for On-neurons) *D*^*sur*^(*x*, *y*, *t*), each being separable in space and time, and round. The spatial kernel displays a center-surround structure determined by axons from retinal ganglion cells described as a difference of two axisymmetric Gaussian functions [85]:

$$D(x,y,t)=D^{cen}(x,y,t)-\;D^{sur}(x,y,t)=\frac{D_{t}^{cen}(t)-B}{\pi \;\sigma_{cen}^{2}}\text{exp}(-\frac{x^{2}+y^{2}}{\sigma_{cen}^{2}})-\frac{D_{t}^{sur}(t)-B}{\pi \;\sigma_{sur}^{2}}\text{exp}(-\frac{x^{2}+y^{2}}{\sigma_{sur}^{2}}).$$ (4)

An example of the LGN neuron’s RF is shown in Fig 1A, **left**. The temporal component is determined by alpha-functions, as in [85], with a coefficient controlling the balance between early and late components of LGN cell responses.

Here $D_{t}^{cen}(t)$ and $D_{t}^{sur}(t)$ are the temporal evolution functions

$$D_{{}_{t}}^{cen}(\tau )=\;t/\tau_{cen}^{2}\;\text{exp}(-t/\tau_{cen})-\;t/\tau_{late}^{2}\;\text{exp}(-t/\tau_{late}).$$ (5)

and

$$D_{{}_{t}}^{cen}(\tau )=\;t/\tau_{sur}^{2}\;\text{exp}(-t/\tau_{sur})-\;t/\tau_{late}^{2}\;\text{exp}(-t/\tau_{late})$$ (6)

with the time constants *τ*_*cen*_, *τ*_*sur*_ and *τ*_*late*_, respectively. The spatial scales of the RF are *σ*_*cen* and_ *σ*_*sur*_. The parameters were: *τ*_*cen*_ = 10*ms*, *τ*_*sur*_ = 20*ms*, *τ*_*late*_ = 64*ms*, *σ*_*cen*_ = 0.3 deg., *σ*_*sur*_ = 1.5 deg. The parameter are from [85,86].

In practice, in order to optimize the memory usage, the convolutions with the time kernels can be substituted by integration of equivalent ordinary differential equations. For example, a variable

$$Q(x,y,t)=\int_{0}^{t}d\tau \;D_{t}^{cen}(x\prime ,y\prime ,\tau )\;S(x\prime ,y\prime ,t-\tau )$$

can be alternatively calculated from the following equation

$$\tau_{cen}^{2}\frac{d^{2}Q}{dt^{2}}+2\tau_{cen}\frac{dQ}{dt}+Q=S(x,y,t).$$ (7)

The *sustained cells* have in 3.5 times slower kinetics [13]. The firing rate $L_{LGN}^{S}(x,y,t)$ is calculated from the Eqs (1)–(6) with the time constant $\tau_{cen}^{S}=3.5\;\tau_{cen}$, $\tau_{sur}^{S}=3.5\;\tau_{sur}$, $\tau_{late}^{S}=3.5\;\tau_{late}$ instead of *τ*_*cen*_, *τ*_*sur*_ and *τ*_*late*_. The *lagged cell* activity is $L_{LGN}^{L}(x,y,t)=L_{LGN}^{}(x,y,t-\delta )$. For the sake of computational efficiency, the activity *L*_*LGN*_ as a function of the delay is calculated approximately as two first terms of the Taylor expansion:

$$L_{LGN}(\tilde{x},\tilde{y},t-\delta (x,y,\tilde{x},\tilde{y}))\approx {\left[L_{LGN}(\tilde{x},\tilde{y},t)-\delta (x,y,\tilde{x},\tilde{y})\frac{\partial L_{LGN}(\tilde{x},\tilde{y},t)}{\partial t}\right]}_{+}$$ (8)

For *Off-neurons*, the firing rate is calculated as $L_{LGN}^{Off}(x,y,t)={\left[-{\tilde{L}}_{LGN}(x,y,t)\right]}_{+}$.

#### Thalamic input to V1

Orientation and direction selectivity is determined by a footprint of the LGN-to-V1 projections as well as by properties of the LGN neurons, which are different for the considered DS mechanisms. The thalamic input is determined as the firing rate *φ*_*Th E*_(*x*, *y*, *t*), which is a convolution of the LGN neuronal activity of the lagged and non-lagged, or transient and sustained, or On- and Off-cells, $L_{LGN}^{L}(x,y,t)$, *L*_*LGN*_(*x*, *y*, *t*),$L_{LGN}^{T}(x,y,t)$,$L_{LGN}^{S}(x,y,t)$,$L_{LGN}^{On}(x,y,t)$,$L_{LGN}^{Off}(x,y,t)$, respectively, with the footprint function $D^{LGN-V1}(x,y,\tilde{x},\tilde{y},i)$, where $i(x,y,\tilde{x},\tilde{y})$ is the index of the LGN neuronal population, which attributes *N*, *NL*, *T*, *S*, *On* or *Off* indexes, respectively:

$$\varphi_{ThE}(x,y,t)=\iint d\tilde{x}d\tilde{y}\;\sum_{i}D^{LGN-V1}(x,y,\tilde{x},\tilde{y},i(x,y,\tilde{x},\tilde{y}))\;L_{LGN}^{i(x,y,\tilde{x},\tilde{y})}(\tilde{x},\tilde{y},t)$$ (9)

A certain form of the footprint function $D^{LGN-V1}(x,y,\tilde{x},\tilde{y},i(x,y,\tilde{x},\tilde{y}))$ depends on the mechanism of DS.

In the L-N and T-S DS models, the footprint of a direction-selective V1 neuron splits into two halves along the axis of elongation, each sending signals from either lagged or nonlagged (transient/sustained) thalamic cells (Fig 1B). For the On-Off model, the footprint of a V1 cell consists of an elongated On zone and a circular Off zone. The width and elongation of the footprints were 0.3 deg. and 0.8 deg., respectively. The shift was 0.6. deg. The neighboring V1 neurons that belong to different orientational hypercolumns have footprints of similar shapes and prefer the same orientation but opposite directions of the stimulus movement. A kernel expression that determines the DS bias is the equation for the thalamic input into V1 neuron, *φ*_*Th E*_(*x*, *y*, *t*), which is given separately for each of the three mechanisms.

#### Lagged and nonlagged cell-based mechanism of DS

For the L-N cell-based mechanism of DS (L-N mechanism), two populations of L-N cells were considered. These populations are equally and homogeneously distributed across the LGN (Fig 1A, **middle**). The L-N cells have round, center-surround receptive fields (Fig 1A, **left**). In this particular version of the model, only On-cells responding to a bright stimulus in the center of RF and being inhibited in the surrounding area of RF are considered. Lagged cell activity is delayed by 40 ms according to estimations from [25].

In accordance with the footprint of the LGN-to-V1 projections (Fig 1A, **middle**), the V1 neurons that prefer a certain direction receive an input signal from the nonlagged cells located on one (right) side of the footprint and from the lagged cells located on the other (left) side, whereas V1 neurons preferring the opposite (downward) direction conversely receive an input signal from the nonlagged cells of the second (left) side and from the lagged cells of the first (right) side.

The footprints of V1 neurons are oriented according to the neuronal positions in pinwheels. The pinwheels with clockwise progression of orientation columns are adjacent to the ones with counterclockwise progression. The pinwheel-centers are distributed on the rectangular grid with the pinwheel radius *R* and indexed by *i*_*PW*_ and *j*_*PW*_. The adjacent columns owing to different pinwheels have the same orientation preferences. The coordinates of the pinwheel-center are *x*_*PW*_ = (2 *i*_*PW*_ − 1)*R*, *y*_*PW*_ = (2 *j*_*PW*_ − 1)*R*. The orientation angle at the point (*x*, *y*) of V1 that belongs to the pinwheel (*i*_PW_, *j*_PW_) is defined as *θ*(*x*, *y*) = arctan((*y* − *y*_*PW*_)/(x − x_*PW*_)). The progression is determined by the factor ${\left(-1\right)}^{i_{PW}+j_{PW}}$. Finally, the input firing rate is

$$\varphi_{ThE}(x,y,t)=\iint d\tilde{x}d\tilde{y}\;D^{LGN-V1}(x,y,\tilde{x},\tilde{y})\;L_{LGN}(\tilde{x},\tilde{y},t-\delta (x,y,\tilde{x},\tilde{y})),$$ (10)

with

$$D^{LGN-V1}(x,y,\tilde{x},\tilde{y})=\frac{1}{\pi \sigma_{pref}\sigma_{orth}}\text{exp}(-\frac{{x^{\prime }}^{2}}{\sigma_{pref}^{2}}-\frac{{y^{\prime }}^{2}}{\sigma_{orth}^{2}}),$$ (11)

where *L*_*LGN*_ is the activity of the LGN neuron ($\tilde{x},\tilde{y}$); $D^{LGN-V1}(x,y,\tilde{x},\tilde{y})$ is the LGN-to-V1 footprint with the width across preferred orientation *σ*_*pref*_ and the width across orthogonal orientation *σ*_*orth*_; $\delta (x,y,\tilde{x},\tilde{y})$ is the delay that determines contributions of either lagged or nonlagged cells. Because the direction preferences are given by a combination of inputs from the L-N neurons according to the footprint (Fig 1B, **left**), DS is determined by the delay $\delta (x,y,\tilde{x},\tilde{y})$, which is formalized as follows:

$$\delta (x,y,\tilde{x},\tilde{y})=40\;ms,\;if\;{\left(-1\right)}^{i_{PW}+j_{PW}}x^{\prime }>0;\;0,otherwise,$$ (12)

$$x^{\prime }=(\tilde{x}-x_{cf})\text{cos}\;\theta -(\tilde{y}-y_{cf})\text{sin}\;\theta$$ (13)

$$y^{\prime }=(\tilde{x}-x_{cf})\text{sin}\;\theta +(\tilde{y}-y_{cf})\text{cos}\;\theta .$$ (14)

Here (*x*_*cf*_, *y*_*cf*_) are the coordinates of the center of the footprint of V1 neuron in LGN, *x*_*cf*_ = *x*_*cf*_(*x*, *y*), *y*_*cf*_ = *y*_*cf*_(*x*, *y*).

#### Transient and sustained cell based mechanism of DS

In the T-S cell based mechanism of DS the input firing rate is

$$\varphi_{ThE}(x,y,t)=\iint d\tilde{x}d\tilde{y}\;D^{LGN-V1}(x,y,\tilde{x},\tilde{y})\;L_{LGN}^{i(x,y,\tilde{x},\tilde{y})}(\tilde{x},\tilde{y},t),$$ (15)

where $i(x,y,\tilde{x},\tilde{y})$ is the index of *T* or *S* neurons, whose firing rates are calculated with different time constants in the temporal kernel of the convolution. It is defined according to the footprint (Fig 1B, **middle**):

$$i(x,y,\tilde{x},\tilde{y})="S",\;if\;{\left(-1\right)}^{i_{PW}+j_{PW}}x^{\prime }>0;\;"T",otherwise.$$ (16)

In this case, $D^{LGN-V1}(x,y,\tilde{x},\tilde{y})$, *x*′ and *y*′ are given by Eqs (11), (13) and (14).

#### On- and Off cell-based mechanism of DS

In the On- and Off cell-based mechanism of DS the input firing rate is

$$\varphi_{ThE}(x,y,t)=\iint d\tilde{x}d\tilde{y}\;(\begin{array}{l} D^{LGN-V1,\;On}(x,y,\tilde{x},\tilde{y})\;L_{LGN}^{On}(\tilde{x},\tilde{y},t)\\ +D^{LGN-V1,\;Off}(x,y,\tilde{x},\tilde{y})\;L_{LGN}^{Off}(\tilde{x},\tilde{y},t) \end{array}),$$ (17)

where the shifted On- and Off-subfields are given by

$$D^{LGN-V1,\;On}(x,y,\tilde{x},\tilde{y})=\frac{1}{\pi \sigma_{pref}\sigma_{orth}}\text{exp}(-\frac{{x^{\prime }}^{2}}{\sigma_{pref}^{2}}-\frac{{y^{\prime }}^{2}}{\sigma_{orth}^{2}}),$$ (18)

$$D^{LGN-V1,\;Off}(x,y,\tilde{x},\tilde{y})=\frac{1}{\pi \sigma_{pref}^{2}}\text{exp}(-\frac{{\hat{x}}^{2}+{\hat{y}}^{2}}{\sigma_{pref}^{2}}),$$ (19)

where

$$\hat{x}=(\tilde{x}-x_{cf}^{Off})\text{cos}\;\theta -(\tilde{y}-y_{cf}^{Off})\text{sin}\;\theta ,$$ (20)

$$\hat{y}=(\tilde{x}-x_{cf}^{Off})\text{sin}\;\theta +(\tilde{y}-y_{cf}^{Off})\text{cos}\;\theta ,$$ (21)

$$x_{cf}^{Off}=x_{cf}-\Delta^{Off}{\left(-1\right)}^{i_{PW}+j_{PW}}\text{cos}\;\theta ,$$ (22)

$$y_{cf}^{Off}=y_{cf}-\Delta^{Off}{\left(-1\right)}^{i_{PW}+j_{PW}}\text{sin}\;\theta ,$$ (23)

with Δ^*Off*^ as the shift of the Off-subfield, and *x*′ and *y*′ given by Eqs (13) and (14).

### Model of V1

The model of V1 cortical area activity is an extension of a previous model describing orientation processing [34]. Neurons in V1 show more complex RFs than LGN cells. The V1 neurons vigorously respond to gratings moving throughout their RFs. It was assumed that most of neurons in V1 are directionally selective [87,88] despite the fact that some neurons are tuned only to orientation [4]. V1 is modeled as a 2-dimensional continuum of neuronal populations. In simulations, the modeled cortical area of the cortex is 1 mm x 1.5 mm and includes six orientation hypercolumns. (Spatial coordinates as arguments of variables are omitted here.) Each point of the cortical continuum contains two neuronal populations, excitatory (E) and inhibitory (I), connected by AMPA (α-amino-3-hydroxy-5-methyl-4-isoxazolepropionic acid), NMDA (N-methyl-d-aspartate), and GABA-A mediated synapses providing recurrent intracortical interactions and AMPA and NMDA for the geniculate input. The strengths of the external connections correspond to the pinwheel architecture, and thus neurons receive inputs in accordance with their orientation and direction preferences. The profile of the intracortical connections is isotropic, i.e., the maximum conductances depend on the distance between the pre- and postsynaptic populations.

According to the definition of a neuronal population [89], neurons of one population located at a given point both receive a common input that comes from presynaptic populations and an individual noise that takes into account any differences in the intrinsic and input parameters of neurons as well as a spontaneous activity of ionic channels. The membrane potentials and ionic channel states of these neurons are dispersed due to the noise, and thus they are distributed in a space of neuronal refractority. Neurons that fire contribute to the population firing rate, which is the output measure of the population activity. The mathematical description of each population is based on the probability density approach [89,90], namely, the CBRD approach [37,38,91,92], where the neurons within each population are distributed according to their refractority states, which are characterized with the time elapsed since the last spike, t*. Single population dynamics are governed by the equations for neuronal density, the mean over noise realizations voltage, and the gating variables. The model for each *E*- and *I*- neuronal populations takes into account two neuronal compartments and a set of voltage-gated ionic currents, including the adaptation currents.

According to the structure of interneuronal connections, the neuronal population firing rate determines the presynaptic firing rate, which in turn controls the dynamics of synaptic conductances. The presynaptic firing rate predetermined by the excitatory population firing rate determines the dynamics of AMPA and NMDA synaptic conductances. The inhibitory population controls GABA conductance. The synaptic conductances are the input signals received by the postsynaptic neuronal populations. The membrane voltage distribution across t* determines the output firing rate and so on.

Model neurons have two compartments: somatic and dendritic. The inhibitory synapses are assumed to be located at the soma, whereas the excitatory synapses are at the dendrites. Because in experiments the synaptic currents are usually measured at the soma, only somatic conductances are calculated in the model. The two-compartment model from [34,93] implicitly solves a reverse voltage-clamp problem, thus estimating the dendritic synaptic current. Each of the somatic AMPA, GABA, and NMDA conductances (the AMPA conductance mediated by the external thalamic terminals is marked by the index with a dash, i.e. *g*_*AMPA*′,*E*_) is calculated with a second order ordinary differential equation [34]. An input function to the equation is the presynaptic firing rate. Equations connecting the somatic firing rate with the presynaptic firing rate are as given in [34], whereas the presynaptic thalamic input has been modified as described. The full CBRD model for interacting adaptive regular spiking pyramidal cells and fast spiking interneurons is given below.

#### CBRD-approach

The conductance-based refractory density approach [37,38,91,94] considers a population of an infinite number of Hodgkin-Huxley-like neurons receiving both a common input and an individual for each neuron noise. In any arbitrary case of transient or steady-state stimulation the firing rate of such population can be quite precisely and computationally effectively calculated by solving a system of equations in partial derivatives, 1-d transport equations. The equations govern an evolution of neuronal states distributed in a phase space of the time elapsed since last spikes, t*. They contain the Hodgkin-Huxley equations for the membrane voltage and gating variables (except sodium ones), parameterized by t*, as well as the equation for the neuronal density in t*-space, *ρ*^*p*^(*t*, t*), where the index of a population *p* substitutes for *E* or *I*. The output characteristic of the population's activity is the firing rate *ν*^*p*^(*t*), which is equal to *ρ*^*p*^ in the state of a spike, t* = 0, i.e. *ν*^*p*^(*t*) ≡ *ρ*^*p*^(*t*, 0).

Basic neurons have 2-compartments with the somatic and dendritic voltages *U*^*p*^(*t*, t*) and $U_{d}^{p}(t,t^{*})$. In comparison with one-compartment model, the extra parameters is the ratio of dendritic to somatic conductances *γ* and the dendritic length. The inhibitory synapses are located at soma, contributing into the somatic synaptic current *I*_*soma*_, whereas the excitatory synapses are at dendrites, determining the dendritic synaptic current *I*_*dendr*_. Due to the construction of the 2-compartment model [93], both type synaptic conductances are imposed to be somatic, in spite of the localization, in order to be compared with experimental whole-cell somatic registrations. Approximations of voltage-gated ionic currents $I_{voltage-gated}^{p}(U^{p},t,t^{*})$ differ for excitatory and inhibitory neurons. Parameterized by t*, the governing equations are as follows:

$$\frac{\partial \rho^{p}}{\partial t}+\frac{\partial \rho^{p}}{\partial t^{*}}=-\rho^{p}H(U^{p},g_{tot}^{p}),$$ (24)

$$C(\frac{\partial U^{p}}{\partial t}+\frac{\partial U^{p}}{\partial t^{*}})=-g_{L}(U^{p}-V_{L})+\frac{2\gamma }{l}\;g_{L}(U_{d}^{p}-U^{p})+I_{voltage-gated}^{p}(U^{p},t,t^{*})+I_{soma}$$ (25)

$$C(\frac{\partial U_{d}^{p}}{\partial t}+\frac{\partial U_{d}^{p}}{\partial t^{*}})=-g_{L}(U_{d}^{p}-V_{rest})-\frac{2}{l}\;g_{L}(U_{d}^{p}-U^{p})+\frac{I_{dendr}}{\gamma },$$ (26)

where $g_{tot}^{p}(t,t^{*})$ is the total conductance including the leak, voltage-gated and synaptic conductance $g_{syn}^{p}(t,U_{}^{p})=g_{AMPA,p}^{}(t)+g_{NMDA,p}^{}(t,U_{}^{p})+g_{GABA,p}^{}(t)$; *l* is the square ratio of the dendritic length to the characteristic length. The somatic and dendritic synaptic currents *I*_*soma*_ and *I*_*dendr*_ are calculated as

$$I_{soma}=g_{GABA,p}(t)(V_{GABA}-U^{p})$$

$$I_{dendr}=(\frac{l\;\tau_{m}^{0}}{2}\frac{d}{dt}+1+\frac{l}{2})(\left(g_{AMPA\prime ,p}(t)+g_{AMPA,p}(t))(V_{AMPA}-U^{p})+g_{NMDA,p}(t)(V_{NMDA}-U^{p}\right)),$$

where the differential operator represents the solution of the reverse problem of dendritic current estimation from somatically registered-like conductances [93]. The synaptic conductance kinetics is estimated from somatic responses to stimulation of presynaptic neuronal population, thus it implicitly accounts not only the kinetics of synaptic channels but also the dendritic and axonal propagation delays. For the dendritic compartment, the differential operator sharpens the transient effect of the channels, thus providing better agreement between somatic postsynaptic currents and potentials. This sharpening affects only glutamatergic channels located on the dendritic compartment.

#### Hazard function

The source term in the Eq (24) is the hazard function *H* which is defined as the probability for a single neuron to generate a spike, if known actual neuron state variables. The approximation of the hazard function *H* has been obtained for the case of white noise [37] and color noise [38] as a function of *U*^*p*^(*t*), ${g_{tot}^{p}(t,t}^{*})$, and parameters of the noise amplitude in the resting state $\sigma_{V}^{0}$, the spike threshold voltage *V*_*th*_, and the ratio of the membrane time constant *τ*_*m*_ = *C*/*g*_*tot*_ to the noise time constant *τ*_*Noise*_, *k* = *τ*_*m*_/*τ*_*Noise*_:

$$H(U,g_{tot}^{p})=A+B,$$ (27)

$$A=\frac{1}{\tau_{m}}e^{0.0061-1.12\;T-0.257\;T^{2}-0.072\;T^{3}-0.0117\;T^{4}}(1-{\left(1+k\right)}^{-0.71+0.0825(T+3)}),$$

$$B=\sqrt{2}\;{\left[-\frac{dT}{dt}\right]}_{+}\sqrt{\frac{2}{\pi }}\;\frac{\text{exp}(-T^{2})}{1+erf\;(T)},\;T=\frac{V_{th}-U^{p}}{\sqrt{2}\;\sigma_{V}}\sqrt{\frac{g_{tot}^{p}}{g_{tot}^{0}}},$$

where *T* is the membrane potential relative to the threshold, scaled by the noise amplitude *σ*_*v*_ which increases with the synaptic conductance as $\sigma_{V}=\sigma_{V}^{0}\sqrt{1+g_{syn}^{p}/g_{tot}^{0}}$; $g_{tot}^{0}$ is the total somatic conductance at rest. The term *A* is the hazard for a neuron to cross the threshold because of noise, derived analytically [37] and approximated by exponential and polynomial for convenience; *B* is the hazard for a neuron to fire because of depolarization due to deterministic drive, i.e. the hazard due to drift in the voltage phase space. Note that the *H*-function is independent of the basic neuron model and does not contain any free parameters or functions for fitting to any particular case. Thus, *H*-function is the same for excitatory and inhibitory populations.

#### Voltage-dependent channels of excitatory neurons

For *E*-neurons, the approximations for the components of the total (except sodium), voltage-gated current ${I_{voltage-gated}^{E}(U}^{E},t,t^{*})=-I_{DR}-I_{A}-I_{M}-I_{AHP}$ are mainly based on the CA1 pyramidal cell model from [95], where instead of full description of calcium dynamics and calcium-dependent potassium currents a cumulative after-spike hyperpolarization (AHP) current, that provides an effect of slow spike timing adaptation [96]. The set of ionic currents includes the voltage-dependent potassium currents *I*_*DR*_ and *I*_*A*_ responsible for spike repolarization, the slow potassium current *I*_*M*_ that contributes to spike frequency adaptation and the potassium current *I*_*AHP*_, implicitly dependent on calcium dynamics and contributing to spike frequency adaptation. Approximating formulas for the currents *I*_*Na*_, *I*_*DR*_, *I*_*A*_ and *I*_*M*_ are taken from [95]; the approximation for *I*_*AHP*_ is given in [96].

The voltage-dependent potassium current *I*_*DR*_:

$$I_{DR}(U^{E},t,t^{*})={\bar{g}}_{DR}\;x(t)\;y(t)\;(U^{E}(t)-V_{K}),$$ (28)

$$\frac{\partial x}{\partial t}+\frac{\partial x}{\partial t^{*}}=\frac{x_{\infty }(U^{E})-x}{\tau_{x}(U^{E})},$$ (29)

$$\frac{\partial y}{\partial t}+\frac{\partial y}{\partial t^{*}}=\frac{y_{\infty }(U^{E})-y}{\tau_{y}(U^{E})}$$ (30)

$$\tau_{x}=1/(a+b)+0.8\;ms;$$

$$x_{\infty }=a/(a+b),$$

$$a=0.17\;\text{exp}((U^{E}+5)\cdot 0.090)\;ms^{-1},$$

$$b=0.17\;\text{exp}(-(U^{E}+5)\cdot 0.022)\;ms^{-1},$$

$$\tau_{y}=300\;ms,$$

$$y_{\infty }=1/(1+\text{exp}((U^{E}+68)\cdot 0.038));$$

The voltage-dependent potassium current *I*_*A*_:

$$I_{A}(U^{E},t,t^{*})={\bar{g}}_{A}\;x^{4}(t)\;y^{3}(t)\;(U^{E}(t)-V_{K}),$$ (31)

$$\frac{\partial x}{\partial t}+\frac{\partial x}{\partial t^{*}}=\frac{x_{\infty }(U^{E})-x}{\tau_{x}(U^{E})},$$ (32)

$$\frac{\partial y}{\partial t}+\frac{\partial y}{\partial t^{*}}=\frac{y_{\infty }(U^{E})-y}{\tau_{y}(U^{E})}$$ (33)

$$\tau_{x}=1/(a_{x}+b_{x})+1\;ms;$$

$$x_{\infty }=a_{x}/(a_{x}+b_{x}),$$

$$a_{x}=0.08\;\text{exp}((U^{E}+41)\cdot 0.089)\;ms^{-1},$$

$$b_{x}=0.08\;\text{exp}(-(U^{E}+41)\cdot 0.016)\;ms^{-1},$$

$$\tau_{y}=1/(a_{y}+b_{y})+2\;ms;$$

$$y_{\infty }=a_{y}/(a_{y}+b_{y}),$$

$$a_{y}=0.04\cdot \text{exp}(-(U^{E}+49)\cdot 0.11)\;ms^{-1},$$

$$b_{y}=0.04\;ms^{-1};$$

The voltage-dependent potassium current *I*_*M*_:

$$I_{M}(U^{E},t,t^{*})={\bar{g}}_{M}\;x^{2}(t)\;y(t)\;(U^{E}(t)-V_{K}),$$ (34)

$$\frac{\partial x}{\partial t}+\frac{\partial x}{\partial t^{*}}=\frac{x_{\infty }(U^{E})-x}{\tau_{x}(U^{E})},$$ (35)

$$\frac{\partial y}{\partial t}+\frac{\partial y}{\partial t^{*}}=\frac{y_{\infty }(U^{E})-y}{\tau_{y}(U^{E})}$$ (36)

$$\tau_{x}=1/(a+b)+8\;ms,$$

$$x_{\infty }=a/(a+b),$$

$$a=0.003\;\text{exp}((U^{E}+45)\cdot 0.135)\;ms^{-1},$$

$$b=0.003\;\text{exp}(-(U^{E}+45)\cdot 0.090)\;ms^{-1},$$

$$\tau_{y}=1000\;ms,$$

$$y_{\infty }=1/(1+\text{exp}((U^{E}+40)/5));$$

The adaptation current *I*_*AHP*_:

$$I_{AHP}(U^{E},t,t^{*})={\bar{g}}_{AHP}\;x(t)\;y(t)\;(U^{E}(t)-V_{K}),$$ (37)

$$\frac{\partial x}{\partial t}+\frac{\partial x}{\partial t^{*}}=\frac{x_{\infty }(U^{E})-x}{\tau_{w}(U^{E})},$$ (38)

$$\frac{\partial y}{\partial t}+\frac{\partial y}{\partial t^{*}}=\frac{y_{\infty }(U^{E})-y}{\tau_{y}(U^{E})}$$ (39)

$$\tau_{w}=2000/(3.3\text{exp}((U^{E}+35)/20)+\text{exp}(-(U^{E}+35)/20))\;ms,$$

$$x_{\infty }=1/(1+\text{exp}(-(U^{E}+35)/4)),$$

$$\tau_{y}=1000\;ms,$$

$$y_{\infty }=1/(1+\text{exp}((U^{E}+40)/5));$$

#### Voltage-dependent channels of interneurons

The model of the fast-spiking single-compartment interneurons taken from [97] reduces the voltage-gated current to an only potassium current

$$I_{voltage-gated}^{I}(U^{I},t,t^{*})={\bar{g}}_{K}\;n^{4}(t)\;(U^{I}(t)-V_{K}),$$ (40)

$$\frac{\partial n}{\partial t}+\frac{\partial n}{\partial t^{*}}=\frac{n_{\infty }(U^{I})-n}{\tau_{n}(U^{I})},$$ (41)

$$\tau_{n}=(0.5+2/(1+\text{exp}(0.045(U^{I}-50)ms;$$

$$n_{\infty }=1/(1+\text{exp}(-0.045(U^{I}+10))).$$

#### Boundary conditions

According to the conservation of the number of neurons in a population, the firing rate is calculated as a sink of neurons from their state t* due to spiking, *ρ*^*p*^(*t*, t*) *H*(*U*^*p*^(*t*, t*), integrated over the whole phase space, i.e.

$$\nu^{p}(t)\equiv \rho^{p}(t,0)=\int_{+0}^{\infty }\rho^{p}(t,t^{*})\;H(U^{p}(t,t^{*}))dt^{*}.$$ (42)

It is the boundary condition for Eq (24).

The spike duration is taken into account by introducing the time interval 0 < t* < Δ*t*_*AP*_ during which the voltage and the gating variables are fixed to their reset values. It defines the boundary conditions for eqs.(A2-A14) at t* = Δ*t*_*AP*_ which are as follows:

$$U^{p}(t,\Delta t_{AP})=V_{reset},$$ (43)

$$U_{d}^{p}(t,\Delta t_{AP})=V_{rest};$$ (44)

$$\;I_{DR}:\;\;x(t,\Delta t_{AP})=0.262,\;\;y(t,\Delta t_{AP})=0.473;$$ (45)

$$\;I_{A}:\;\;x(t,\Delta t_{AP})=0.743,\;\;y(t,\Delta t_{AP})=0.691.$$ (46)

The reset values for the fast gating variables in Eqs (45) and (46) were obtained with the basic single neuron model. With a rather arbitrary input providing a spike, these values were measured at the moment of a voltage maximum at the spike. The reset level for each slow conductance in the CBRD model was calculated as a sum of its value at a peak of spike-release distribution in the t*-space and an increment at spike:

$$\;I_{M}:\;\;x(t,\Delta t_{AP})=x(t,t^{**})+0.175\;(1-x(t,t^{**})),\;y(t,\Delta t_{AP})=y(t,t^{**})-0.003\;y(t,t^{**});$$ (47)

$$\;I_{AHP}:\;\;x(t,\Delta t_{AP})=x(t,t^{**})+0.018\;(1-x(t,t^{**})),\;y(t,\Delta t_{AP})=y(t,t^{**})-0.003\;y(t,t^{**});$$ (48)

where t** is such that

$$\rho (t,t^{**})\;H(t,t^{**})=\underset{\;0<\;t^{*}<+\infty }{\text{max}}\;\rho (t,t^{*})\;H(t,t^{*}).$$

The increment values for the slow gating variables in Eqs (47) and (48) were also measured at a single spike of the single neuron model.

Parameters of basic neurons are as follows:

$${\bar{g}}_{DR}=0.76\;\mu S/cm^{2},\;\;{\bar{g}}_{A}=4.36\;\mu S/cm^{2},$$

$${\bar{g}}_{M}=0.76\;\mu S/cm^{2},\;\;{\bar{g}}_{AHP}=0.6\;\mu S/cm^{2},$$

$$C=0.7\;\mu F/cm^{2},\;\;\tau_{m}^{0}=C/g_{tot}^{0}=14.4\;ms,\;(g_{L}=0.048\;\mu S/cm^{2}),$$

$$V_{th}(t^{*})=(-40+50\text{exp}(-t^{*}/10\;ms)\;mV,$$

$$V_{reset}=-40\;mV,\;\Delta t_{AP}=1.5\;ms,$$

$$\gamma =2.85,\;\sigma_{V}^{0}=6\;mV,$$

$$S=10^{-4}\;cm^{2}.$$

Here *S* is the membrane area. The dependence of *V*_*th*_(t*) is taken from a full single neuron model [37], allowing to take into account the effect of sodium channel inactivation on the threshold dynamics [98]. *σ*_*ν*_ is the noise amplitude meaning the dispersion of individual neuron’s voltage fluctuations in a stationary state. Its scaling with *g*_*syn*_ approximately reflects the fact of the synaptic noise increase with the increase of mean synaptic drive [99]. Stochastic input to *E2*-neurons *I*_*noise*_ was modeled as Ornstein-Uhlenbeck process with the time correlation 10 ms and the dispersion 20pA for the ID-regime simulation and 40pA for the IID-regime simulation.

The equations for the input synaptic conductances are given below, as well as the values of the reversal potentials. When calculating the dynamics of a neural population, the integration of Eqs (25), (26) and (28)–(39) determines the evolution of the distribution of voltage *U*^*E*^ across t*. Then, the effect of crossing the threshold and the diffusion due to noise are taken into account by *H*-function, Eq (27), substituted into the equation for neuronal density, Eq (24). The integral Eq (40) results in the output firing rate *ν*^*E*^(*t*).

#### Lognormal distribution of synaptic weights within each population

The CBRD-approach is generalized to the case of lognormal distribution of synaptic weights within each *p*-population [100]. In this case, instead of equal total synaptic current, neurons receive lognormally distributed current. For the current scaled by its mean across the distribution, *η*, the distribution is

$$\psi (\eta )=\frac{\text{exp}(-{\left(\text{ln}\;\eta \right)}^{2}/(2\;\sigma_{LN}^{2}))}{\sqrt{2\pi }\;\sigma_{LN}\;\eta }$$ (49)

The membrane potential of neurons parameterized with *η*, $U_{\eta }^{p}$, can be found as

$$U_{\eta }^{p}(t,t^{*})=(U^{p}(t,t^{*})-U_{free}^{p}(t^{*}))\;x+U_{free}^{p}(t^{*}),$$ (50)

where $U_{free}^{p}(t^{*})$ is the unperturbed potential defined for zero synaptic input.

The density of neurons parameterized by *η* and distributed in the phase space t* is denoted as $\rho_{\eta }^{p}(t,t^{*})$. Calculation of $\rho_{\eta }^{p}(t,t^{*})$ requires solving of a continuum of Eq (1) (or Eq (22)) for $\rho_{\eta }^{p}$ instead of *ρ*^*p*^ with $H(U_{\eta }^{p},dU_{\eta }^{p}/dt)$. The output firing rate is defined as

$$\nu^{p}(t)=\int_{0}^{\infty }\rho_{\eta }^{p}(t,0)\;\psi (\eta )\;d\eta$$ (51)

In numerical simulations, we set the parameter of the lognormal distribution *σ*_*LN*_ = 0.75 and discretized the *η*-space by 15 intervals.

Mean somatic and dendritic membrane potentials are calculated as follows:

$$\bar{U}(t)=\int_{0}^{\infty }U(t,t^{*})\;\int_{0}^{\infty }\;\rho_{\eta }(t,t^{*})\;\psi (\eta )\;d\eta \;dt^{*}$$ (52)

and

$$\bar{U_{d}}(t)=\int_{0}^{\infty }U_{d}(t,t^{*})\;\int_{0}^{\infty }\;\rho_{\eta }(t,t^{*})\;\psi (\eta )\;d\eta \;dt^{*}.$$ (53)

#### Approximation of synaptic conductances

The synaptic conductances are described with the second-order differential equations [34] as follows:

$$g_{AMPA\prime ,p}(t)={\bar{g}}_{AMPA\prime ,p}\;m_{AMPA\prime ,p}(t)\;,g_{AMPA,p}(t)={\bar{g}}_{AMPA,p}\;m_{AMPA,p}(t)\;,$$ (54)

$$g_{NMDA,p}(t,U^{p})={\bar{g}}_{NMDA,p}\;f_{NMDA}(U^{p}(t))\;m_{NMDA,p}(t),$$ (55)

$$f_{NMDA}(V)=1/(1+Mg/3.57\;\text{exp}(-0.062\;V)),$$

$$g_{GABA,p}(t)={\bar{g}}_{GABA,p}\;m_{GABA,p}(t),$$ (56)

*M*_*g*_ is the magnesium (Mg^2+^) concentration in mM; *m*_*s*,*p*_(*t*) is the non-dimensional synaptic conductance which is approximated by the second order ordinary differential equation:

$$(\tau_{r}^{s,p}\tau_{d}^{s,p}\frac{d^{2}}{dt^{2}}+(\tau_{r}^{s,p}+\tau_{d}^{s,p})\frac{d}{dt}+1)\;m_{s,p}(t)=\tau^{s,p}(1-m_{s,p}(t))\;\varphi_{i,p}(t),$$ (57)

$$\tau^{s,p}=(\tau_{r}^{s,p}-\tau_{d}^{s,p})/({\left(\tau_{d}^{s,p}/\tau_{r}^{s,p}\right)}^{\tau_{d}^{s,p}/(\tau_{r}^{s,p}-\tau_{d}^{s,p})}-{\left(\tau_{d}^{s,p}/\tau_{r}^{s,p}\right)}^{\tau_{r}^{s,p}/(\tau_{r}^{s,p}-\tau_{d}^{s,p})}),$$ (58)

$$if\;\tau_{r}^{s,p}\neq \tau_{d}^{s,p},$$

$$\tau_{r}^{s,j}e,\;otherwise.$$

Here *φ*_*i*,*p*_ is the presynaptic firing rate determined by axons of the population *i* on the postsynaptic population *p*. In neglect of spatial propagation and temporal delays the presynaptic firing rate is equivalent to the somatic firing rate, i.e.*φ*_*i*,*p*_ ≡ *ν*_*i*_. The index *s* is the synapse type, *s* = *AMPA*, *GABA* or *NMDA*; the index *i* = *Th* means thalamic input for *s* = *AMPA*′; *i* = *E* for *s* = *AMPA* or *NMDA*; and *i* = *I* for *s* = *GABA*; ${\bar{g}}_{s,p}$ is the maximum conductance, $\tau_{r}^{s,p}$ and $\tau_{d}^{s,p}$ are the rise and decay time constants. We imply that the synaptic time constants are estimated from the somatic responses to the stimulation of a presynaptic neuronal population, thus these time constants characterize not only synaptic channel kinetics but the dendritic and axonal propagation delays as well. The time scale *τ*^*s*,*p*^ is chosen in the form of Eq (56) in order to provide independence of the maximum of *g*_*s*,*p*_(*t*) on $\tau_{r}^{s,p}$ and $\tau_{d}^{s,p}$, when *g*_*s*,*p*_(*t*) is evoked by a short pulse of *φ*_*i*,*p*_(*t*).

The parameter values were as follows: ${\bar{g}}_{AMPA\prime ,I}=0$, ${\bar{g}}_{AMPA\prime ,E}={\bar{g}}_{AMPA,E}={\bar{g}}_{AMPA,I}=0.4$ mS/cm^2^, ${\bar{g}}_{NMDA,E}={\bar{g}}_{NMDA,I}=1.6$ mS/cm^2^, ${\bar{g}}_{GABA,E}=1.2$ mS/cm^2^, ${\bar{g}}_{GABA,I}=0.2$ mS/cm^2^, *V*_*AMPA*_ = *V*_*NMDA*_ = 0, *V*_*GABA*_ = −77mV, *Mg* = 2 mM, $\tau_{r}^{AMPA,E}=\tau_{r}^{AMPA,I}=1.7$ ms, $\tau_{d}^{AMPA,E}=\tau_{d}^{AMPA,I}=8.3$ ms, $\tau_{r}^{NMDA,E}=\tau_{r}^{NMDA,I}=6.7$ ms, $\tau_{d}^{NMDA,E}=\tau_{d}^{NMDA,I}=100$ ms, $\tau_{r}^{GABA,E}=\tau_{r}^{GABA,I}=0.5$ ms, $\tau_{d}^{GABA,E}=\tau_{d}^{GABA,I}=20$ ms.

#### Representative neurons

Representative neurons of each of the populations were modeled according with the basic single neuron model with the same synaptic inputs as for the populations. The activity of the representative neurons does not affect the network. The representative neuron model is described by the equations for the membrane voltage, Eqs (25), (26) and (28)–(39) for *E*-population, and Eqs (24), (25), (40) and (41) for *I*-population, where the sum of partial derivatives were substituted by the total derivative in time *t*, and the sodium current was explicitly present in the right-hand part of Eq (25). The sodium current dependent on voltage *U* was approximated by the 4-state Markov model [95]:

$$I_{Na}(t)={\bar{g}}_{Na}\;x_{1}(t)(U(t)-V_{Na}),$$

$$x_{1}+x_{2}+x_{3}+x_{4}=1,$$

$$\frac{dx_{i}}{dt}=\sum_{j=0,\;j\neq i}^{4}A_{j,i}x_{j}-x_{i}\sum_{j=0,\;j\neq i}^{4}A_{i,j},\;i=1,2,3$$

$$A_{1,2}=3\;ms^{-1},\;A_{1,3}=f_{1}^{1,3}(U),\;\;A_{1,4}=f_{1}^{1,4}(U),$$

$$A_{2,1}=0,\;A_{2,3}=f_{2}^{2,3}(U),\;A_{2,4}=0,$$

$$A_{3,1}=f_{1}^{3,1}(U),\;\;A_{3,2}=0,\;\;A_{3,4}=f_{2}^{3,4}(U),$$

$$A_{4,1}=f_{1}^{4,1}(U),\;\;A_{4,2}=0,\;\;A_{4,3}=0$$

$$f_{1}^{i,j}(U)={\left\{\tau_{min}^{i,j}+1/\text{exp}(U-V_{1/2}^{i,j}k^{i,j})\right\}}^{-1},$$

$$f_{2}^{i,j}(U)={\left\{\tau_{min}^{i,j}+{\left[{\left(\tau_{max}^{i,j}-\tau_{min}^{i,j}\right)}^{-1}+\text{exp}(U-V_{1/2}^{i,j}k^{i,j})\right]}^{-1}\right\}}^{-1},$$

$$\tau_{min}^{1,3}=1/3\;ms,\;\;V_{1/2}^{1,3}=-51\;mV,\;\;k^{1,3}=-2\;mV,$$

$$\tau_{min}^{1,4}=1/3\;ms,\;\;V_{1/2}^{1,4}=-57\;mV,\;\;k^{1,4}=-2\;mV,$$

$$\tau_{min}^{2,3}=1\;ms,\;V_{1/2}^{2,3}=-53\;mV,\;k^{2,3}=-1\;mV,\;\;\tau_{max}^{2,3}=100\;ms,$$

$$\tau_{min}^{3,1}=1/3\;ms,\;V_{1/2}^{3,1}=-42\;mV,\;\;k^{3,1}=1\;mV,$$

$$\tau_{min}^{3,4}=1\;ms,\;V_{1/2}^{3,4}=-60\;mV,\;k^{3,4}=-1\;mV,\;\;\tau_{max}^{3,4}=100\;ms,$$

$$\tau_{min}^{4,1}=1/3\;ms,\;\;V_{1/2}^{4,1}=-51\;mV,\;\;k^{4,1}=1\;mV.$$

#### Numerical approach for CBRD model

The transport equations with the independent variables *t* and t* are solved with numerical scheme constructed in the framework of the Lagrangian description. The semi-infinite t*-space is bounded by the interval [0, *B*] and discretized by *N* intervals. Each *i*-indexed interval is represented by a non-spiking probe neuron, initially located at $t_{i}^{*}$. Each probe neuron *i* represents a certain fraction of a population. The states of the probe neurons (the voltage and gating variables) and the neuronal density attributed to probe neurons evolve according to the main transport equations with the total derivative in time in the left-hand part. Their t*-coordinates increase up to *B*. If a probe neuron reaches t* = *B*, then its t*-coordinate is renewed to 0, their potential and gating variables are replaced or incremented in accordance with the boundary conditions. The neuronal density at t* = 0 is equal to the flux *ρH* accumulated during the time *B*/*N*. In the present study, this approach has been applied with the parameters *B*^*E*^ = 100ms, *N*^*E*^ = 100, *B*^*I*^ = 40ms, *N*^*I*^ = 50.

#### Spatial connections

The horizontal cortical connections are supposed to be local and isotropic. In contrast to [98], the patchy connections have been neglected. They are determined by the relations between the somatic rates *ν*^*i*^ and presynaptic firing rates *φ*_*i*,*j*_, where *i* and *j* are the indexes of the pre- and postsynaptic populations, respectively. In the case of 2-d geometry, all variables depend on the spatial coordinates *x* and *y*, oriented along the surface of the cortex. A gaussian profile of the strengths of the connections is assumed, i.e.

$$\phi_{i,j}(t,x,y)=\iint \nu^{i}(t,x\prime ,y\prime )\;e^{-({\left(x-x\prime \right)}^{2}+{\left(y-y\prime \right)}^{2})/d_{i,j}^{2}}dx\prime \;dy\prime /\iint \;e^{-({\left(x-x\prime \right)}^{2}+{\left(y-y\prime \right)}^{2})/d_{i,j}^{2}}dx\prime \;dy\prime$$

where *d*_*i*,*j*_ is the characteristic length. The parameters were as follows: *d*_*E*,*E*_ = 100*μm*, *d*_*E*,*I*_ = 500*μm*, *d*_*I*,*E*_ = 200*μm* and *d*_*I*,*I*_ = 100*μm*, which roughly correspond to electrophysiological paired recordings estimations [101]. Cortex region was 1 mm×1,4 mm, containing 2×3 regularly distributed pinwheels. Numerical parameters: time step 0.1ms, spatial grid 24×36.

The software code written in Delphi and the compiled program “Brain” are available from http://www.ioffe.ru/CompPhysLab/MyPrograms/Brain/Brain.zip.

### Modeling of V1 silencing and recordings from single neurons

To reveal the contribution of LGN-to-V1 projections into DS, Lien and Scanziani [13,41] optogenetically inhibited the visual cortex of mice with interneurons expressing channelrhodopsin. Monochrome light led to the activation of interneurons and the subsequent inhibition of their target neurons, including the excitatory neurons in layer 4. In this way, the contribution of cortico-cortical interconnections was minimized, thus revealing the contribution of LGN-to-V1 connections. In this model, we substituted the effect of light by the effect of the depolarizing current 100 pA applied to the interneurons.

To register the behavior of a single representative neuron, we simulated the neuron with the basic Hodgkin-Huxley-like model that was used for the construction of the CBRD model. Because in the cortex each single neuron receives synaptic inputs determined by the activity of neuronal populations, the representative neuron was simulated with synaptic conductances obtained from the equations of the population activity. In addition, the neuron can be controlled by a patch-clamp electrode that injects a current in the current-clamp mode or holds the membrane potential in the voltage-clamp mode. For instance, experimental registrations by Lien and Scanziani [13,41] were done with the voltage clamped at the level of the GABAergic channel reversal potential, thus measuring EPSC. In simulations, it is equal to -77mV. Because the representative neuron is simply used to observe the population dynamics, it does not affect the network. Locations of the representative neurons are marked with white dots in Figs 3C, 4A and 4B. For the sake of statistical measurements of the ratio of the charges supplied with the EPSC for intact versus inhibited cortex *Q*_*control*_/*Q*_*inhibited*_ (Fig 8), we simulated patch-clamp recordings in the voltage-clamp mode in all nodes of the spatial grid. Specifically, for each of the DS models we: (i) calculated EPSCs in all nodes of the spatial grid, (ii) calculated *Q*_*control*_ and *Q*_*inhibited*_ by integrating over the interval from 0 to 1600ms the EPSCs with the subtracted background level before the stimulation (-135 and -125pA for the intact and inhibited cortex, respectively), and (iii) calculated the mean value and the dispersion of *Q*_*control*_/*Q*_*inhibited*_ across all nodes with the same preferred orientation as in the site shown in Fig 3C.

### Data analysis

For each point of the computational domain the field of the excitatory population firing rate *ν*^*E*^(*t*, *x*, *y*) and the membrane potential ${\overset{-}{U}}^{E}(t,x,y)$ were averaged over the time interval from 600 to 1600ms since the stimulus onset. Based on modulations of these signals, the field of the preferred directions was found. At each point, the direction selectivity index (DSI) was calculated as in [13]: DSI = (Pref − Nonpref)/Pref, where Pref and Nonpref are the amplitudes of the modulated component of the response (firing rate or voltage) to gratings of the preferred and opposite directions, The mean DSIs were calculated by averaging over the entire computational domain.

The charge Q was calculated as an integral of *EPSC*(*t*) − *EPSC*_0_ over the time period from 0 to 1600ms, where *EPSC*_0_ is the steady-state response to the initial gray screen. With the basic parameters it was equal to -135 and -125pA for the intact and inhibited cortex, respectively.
